# Not all grammar errors are equally noticed: error detection of naturally occurring errors and implications for eye-tracking models of everyday texts

**DOI:** 10.3389/fpsyg.2023.1124227

**Published:** 2023-07-13

**Authors:** Katrine Falcon Søby, Byurakn Ishkhanyan, Line Burholt Kristensen

**Affiliations:** Department of Nordic Studies and Linguistics, University of Copenhagen, Copenhagen, Denmark

**Keywords:** grammar, error detection, proofreading, production, processing models

## Abstract

Grammar errors are a natural part of everyday written communication. They are not a uniform group, but vary from morphological errors to ungrammatical word order and involve different types of word classes. In this study, we examine whether some types of naturally occurring errors attract more attention than others during reading, measured by detection rates. Data from 211 Danish high school students were included in the analysis. They each read texts containing different types of errors: syntactic errors (verb-third word order), morphological agreement errors (verb conjugations; gender mismatches in NPs) and orthographic errors. Participants were asked to underline all errors they detected while reading for comprehension. We examined whether there was a link between the type of errors that participants did not detect, the type of errors which they produce themselves (as measured in a subsequent grammar quiz), and the type of errors that are typical of high school students in general (based on error rates in a corpus). If an error is infrequent in production, it may cause a larger surprisal effect and be more attended to. For the three subtypes of grammar errors (V3 word order, verb errors, NP errors), corpus error rates predicted detection rates for most conditions. Yet, frequency was not the only possible explanation, as phonological similarity to the correct form is entangled with error frequency. Explicit grammatical awareness also played a role. The more correct answers participants had in the grammar tasks in the quiz, the more errors they detected. Finally, we found that the more annoyed with language errors participants reported to be, the more errors they detected. Our study did not measure eye movements, but the differences in error detection patterns point to shortcomings of existing eye-tracking models. Understanding the factors that govern attention and reaction to everyday grammar errors is crucial to developing robust eye-tracking processing models which can accommodate non-standard variation. Based on our results, we give our recommendations for current and future processing models.

## 1. Introduction

Everyday texts, whether it is an email to a colleague or a high school essay, are rarely edited. Such texts often contain grammar errors like anomalous use of word order and lack of agreement between verb and subject (Lunsford and Lunsford, 2008). Attention to these errors is not uniform. In some cases, readers react to the error. In other cases, the error goes by unnoticed. This variation in the reader’s attention and response to errors poses a challenge to existing models of eye movement control in reading, such as E-Z Reader (Reichle et al., 2003) and SWIFT (Engbert et al., 2005). Enhancing our understanding of the factors that govern attention and reaction to everyday grammar errors is necessary for developing robust models of eye movement control (Søby et al., 2023). We need models that take into account variation in the type of naturally occurring grammar anomalies that occur in non-standard language and variation in the reader’s grammatical awareness and proficiency, as both these factors may modulate attention and eye movements.

Differential attention to language errors has been examined in previous studies using different methods. Proofreading studies show that attention is not equally distributed between different types of language errors (Hacker et al., 1994; Shafto, 2015). Typos like *toujousr* for *toujours* attract more attention than grammar errors, which again attract more attention than orthographic errors with phonological similarity to the correct form, e.g., *essentiellemment* for *essentiellement* (Larigauderie et al., 2020).

Change blindness studies also provide evidence for differential attention allocation. In this paradigm, a participant reads two almost identical sentences, one after another, and responds to whether the two sentences are identical or not. Only one word is changed from the first display of the sentence to the second. Change blindness studies show that readers attend more to changes in lexical elements (e.g., full verbs and demonstrative pronouns) than to changes in grammatical elements (e.g., auxiliaries and articles; Christensen et al., 2021) and that readers attend more to changes in focused words than in non-focused words (Sturt et al., 2004).

Across EEG and eye-tracking studies, the difference between syntactic and semantic/pragmatic anomalies is well-documented (Ainsworth-Darnell et al., 1998; Ni et al., 1998; Braze et al., 2002; Hahne and Friederici, 2002; Hagoort, 2003). Grammar errors, however, are usually treated as a homogenous group, although grammar errors involve various subtypes (word order errors, verb agreement errors, gender mismatch errors etc.) which are not necessarily noticed to the same degree or not necessarily processed in the same way. With the present study we ask, if sensitivity to different kinds of grammar errors differs too, and what the consequences are for existing models of eye movement control in reading.

Using an error detection paradigm, we study the differences in attention to different types of naturally occurring grammar errors in written Danish. Some error types involve attention to confusion of large elements (e.g., word order errors), while others involve smaller segments at the level of words, suffixes and letters. Some errors appear initially in a sentence. Other errors have a medial or final position. Some grammar errors have phonological similarity with the correct form, and others are distinct. Many of these factors co-vary in naturally occurring errors and cannot be completely disentangled. In our study, we focus on how error type, error frequency in written production and phonological similarity to the correct form affect readers’ perception of and attention to grammar errors in Danish. For word order errors, we also consider the position of the misplaced word in the sentence.

Previous studies of writers’ spelling accuracy show that exposure to incorrectly spelled words tends to negatively influence later spelling accuracy for those same words (Jacoby and Hollingshead, 1990). Building on these findings, we propose that previous exposure to specific types of incorrectly inflected or misplaced words may also affect attention to this specific type of grammar errors during reading. We also examine the relationship between the type of errors that young readers tend to overlook in texts, the type of errors these young readers produce themselves (when performing a grammar quiz), and the type of errors that are typical of their age group in general (based on corpus studies of naturally occurring texts). Some grammar errors in our study represent types of errors that frequently occur in Danish high school essays. Other errors are less typical of high school students, but characteristic of L2 learners of Danish. We investigate if these typical L2 grammar errors attract more attention than the grammar errors typical of high school students. Our expectation is that attention to a specific type of grammar error is not only a matter of the reader’s explicit grammar awareness (as measured in the grammar quiz), but also of whether the specific type of error is common in everyday texts by native speakers. If a specific type is frequent among the peers of the reader, the reader may have more exposure to this type of error and a mental representation of the error. The reader may therefore find it less striking and be less likely to detect it than errors that are infrequent in texts written by peers.

## 2. Background

Our error detection study does not involve eye-tracking data, but in combination with insights from previous eye-tracking studies on processing of grammar errors, it can address shortcomings in current models of eye movement control during reading. In this section, we present previous eye-tracking studies on processing of grammar errors (section 2.1), and describe the role of grammar errors in existing models of eye movement control in reading (section 2.2). In section 2.3, we describe the error detection paradigm, and how this may contribute to research in attention during reading. We also present the error types chosen for this study. Finally, in section 2.4, we provide an overview of the main factors presumed to influence attention to errors.

### 2.1. Previous eye-tracking studies on processing of grammar errors

Previous eye-tracking studies of grammar errors differ with respect to language, error types, purpose of the study, and the included reading measurements. Therefore, the findings cannot be easily summarized.

First, the eye-tracking studies have been conducted in different languages (English, Hebrew and Norwegian), making it difficult to compare across studies. For example, it is difficult to compare Hebrew subject-verb gender agreement to Norwegian word order.

Second, the ungrammatical items are very different, ranging from word order errors such as *The white* *was cat* *big* (Huang and Staub, 2021), Norwegian *ASV word order instead of AVS (Søby et al., 2023) to various morphosyntactic agreement errors such as gender agreement (Deutsch and Bentin, 2001; Dank et al., 2015), subject-verb agreement (Pearlmutter et al., 1999; Lim and Christianson, 2015) or modals followed by a progressive form, e.g., *It seems that the cats will not usually *eating* the food we put on the porch* (Ni et al., 1998), and/or a past tense form (Braze et al., 2002).

Third, previous studies have had different reasons for including ungrammatical items. Their experimental contrasts differ and their results can be difficult to compare. Huang and Staub (2021) examined failure to notice transposition errors to enter a debate about serial vs. parallel processing. Other studies focus on the differences between pragmatic and syntactic processing (Ni et al., 1998; Braze et al., 2002), or the interrelation between semantic and syntactic factors during processing of agreement in Hebrew (Deutsch and Bentin, 2001). Other studies again have investigated the attraction phenomenon, i.e., when a word erroneously agrees with a local distractor noun instead of the head noun, e.g., *The key to the cabinets *were* rusty from many years of disuse* (Pearlmutter et al., 1999), both in English (Lim and Christianson, 2015) and for subject-predicate agreement in Hebrew (Dank et al., 2015).

Finally, the studies use different reading measurements. While some measure very early effects, such as first fixation duration (Deutsch and Bentin, 2001; Braze et al., 2002; Dank et al., 2015; Lim and Christianson, 2015; Huang and Staub, 2021; Søby et al., 2023); others do not (Ni et al., 1998; Pearlmutter et al., 1999).

Taking these reservations into account, it seems that the different types of grammar errors elicit similar responses in participants’ eye movements across languages, with similar time courses. Most of the studies find more regressions out from the error, meaning that participants respond immediately. Most studies also find increased reading times, but the time course varies (see Søby et al., 2023). Very early effects are found on first fixation duration by Deutsch and Bentin (2001), Dank et al. (2015), Huang and Staub (2021), and partly by Søby et al. (2023). Other studies only find increased total durations on the critical region (Pearlmutter et al., 1999) or no reading time effects at all (Ni et al., 1998). Typically the effects of ungrammaticality quickly disappears, either in the critical or subsequent regions.

Only one of the previous eye-tracking studies has explicitly examined whether readers perceived the ungrammatical items as errors or not. Huang and Staub (2021) used readers’ grammaticality judgments of each sentence to distinguish between detected and undetected errors. None of the studies have made direct comparisons between different types of grammar errors to examine whether participants elicit stronger or different reactions to some errors than others. Therefore, little is known about the factors that govern attention and reaction to different types of grammar errors. Furthermore, the ecological validity of grammar errors have not been the focus of previous studies. Errors such as transposed words are constructed for the purpose of the experiment, but infrequent in natural language, and therefore may not reflect reading processes for naturally occurring language. Understanding the factors that govern attention and reaction to different types of naturally occurring errors is a necessary prerequisite when developing robust eye-tracking models for reading everyday texts (Søby et al., 2023).

### 2.2. The role of grammar errors in existing models of eye movement control in reading

Attention to, and processing of, grammar errors have not been a focal point in existing models of eye movement control in reading. Existing models can be divided into two types. *Serial-attention* models share the assumptions that attention is allocated serially, and only to one word at a time, while *attention-gradient* models assume that attention is allocated as a gradient, i.e., to multiple words at a time (Warren, 2011, p. 919). The major models are the influential E-Z Reader (Reichle et al., 2003, 2009; Reichle, 2011), a serial-attention model, and SWIFT, an attention-gradient model (Engbert et al., 2005; Engbert and Kliegl, 2011). Serial-attention models are furthermore described as *cognitive control* models, because they assume that “lexical processing is the ‘engine’ that determines when the eyes will move from one word to the next during reading” (Reichle, 2011, p. 768), in contrast to models like SWIFT, in which cognition is assumed to play a reduced role for eye movements. For example, the signal to move the eyes forward in SWIFT is provided by an autonomous random timer.

Both E-Z Reader and SWIFT account for effects of lexical processing on eye movements, but a widely acknowledged shortcoming of both models is that they cannot account for effects of higher-level language processing on eye movements (Clifton and Staub, 2011; Warren, 2011). The issue has not been addressed in SWIFT, but for E-Z Reader, Reichle et al. (2009) added a post-lexical integration stage, which is assumed to reflect all of the postlexical processing that is required to integrate a word, *n*, into the higher-level representations which readers construct online. As exemplified by Reichle et al. (2009, p. 5f), this could be to link word *n* into a syntactic structure, to generate a context-appropriate semantic representation, and to incorporate its meaning into a discourse model. Reichle et al. (2009, p. 6) state that “the integration stage […] is a placeholder for a deeper theory of postlexical language processing during reading. Our goal in including this stage is therefore quite modest: to provide a tentative account of how […] postlexical variables might affect readers’ eye movements.”

In E-Z Reader ver. 10 (Reichle et al., 2009; Reichle, 2011), lexical processing of a word takes place in two stages. First, the early stage of lexical processing (or word identification), also known as *L_1_* or the *familiarity check,* takes place. This stage corresponds to the identification of the orthographic form of the word, assuming that “this is not full lexical access, as the phonological and semantic forms of the word are not yet fully activated” (Reichle et al., 2003, p. 452). When completed, i.e., when the feeling of familiarity concerning the word exceeds a threshold corresponding to the familiarity check, it triggers the initiation of the programming of a saccade to move the eyes to the next word (Reichle, 2011). The time required to finish the familiarity check depends on the frequency of a word and its cloze probability, defined as the proportion of subjects who are able to guess word *n*, when shown the rest of the sentence (Reichle et al., 2009:3). This predicts that frequent and/or predictable words are processed faster than infrequent and/or unpredictable words (Reichle, 2011). We assume that the same reasoning applies to frequent and/or predictable errors, but the E-Z Reader model does not explicitly account for input with frequent vs. infrequent errors.

The later stage of lexical processing (*L_2_*) involves the identification of the word’s phonological and/or semantic forms, to enable additional linguistic processing (Reichle et al., 2003). This stage corresponds to what is typically referred to as *lexical access,* and with the completion of lexical access, attention shifts to the next word, which can now be processed. Simultaneously, post-lexical processing (i.e., integration) starts on the identified word. This post-lexical processing corresponds to the minimal amount of processing necessary to continue to move attention (and the eyes) forward through the text (Reichle, 2011, p. 776). In most cases, integration is completed without difficulty, meaning that post-lexical processing only has minimal effect on readers’ eye movements. Reichle et al. (2009, p. 6) assume that complete incremental post-lexical processing is not always necessary and does not always occur, which is broadly consistent with the “good enough” view of language processing (Ferreira and Patson, 2007). However, integration difficulty may occur. When integration fails, it causes the eyes and attention to pause and/or move backwards (Reichle, 2011). Integration failures happen by default when word *n* + 1 is identified before word *n* is integrated. Rapid integration failure can happen due to severe semantic or syntactic violations (Reichle et al., 2009). If the integration of *n* fails rapidly, the forward saccade to *n* + 1 is canceled, which results in a pause (increasing first fixation duration and gaze duration) and/or a refixation (increasing gaze duration) or an interword regression (Reichle et al., 2009). If the integration failure of *n* takes place after the eyes have moved to *n +* 1, i.e., fails more slowly, a regressive eye movement is made (Clifton and Staub, 2011, p. 904). Thus, the model predicts that problems with integration can have very rapid effects, influencing first-fixation duration on the word that is being integrated. This, however, only happens when the integration failure occurs before the labile stage of saccadic programming (i.e., the stage which can be canceled) to move the eyes forward in the text has completed (Reichle et al., 2009).

The assumption that contextual information (besides cloze probability) only affects postlexical integration is challenged by studies of parafoveal processing, i.e., processing of upcoming words that have been attended, but not yet fixated (Warren, 2011). For example, Veldre and Andrews (2018) used the gaze-contingent boundary paradigm to assess whether parafoveal processing of a word contributes to its subsequent identification. In this paradigm, a target word in a sentence is replaced with another word, until the reader’s eyes cross an invisible boundary (e.g., before the space to the left of the target word), after which the word is changed back to the target word. Veldre and Andrews (2018) conducted two experiments, in which they compared contextually plausible previews (which either contained a morphosyntactic agreement violation or not) to implausible previews (either containing a syntactic word class violation or not). The plausible previews were not predictable from the sentence context, as measured in a cloze task. Veldre and Andrews (2018) found that the contextual plausibility and grammatical correctness of an upcoming word can affect processing, early enough to affect skipping of that word. According to the authors, the plausibility effects on skipping rates are unlikely to be reconciled with E-Z Reader’s current post-lexical integration mechanisms.^1^

Furthermore, the E-Z Reader model does not address what happens when readers encounter other types of misspellings or grammar errors, besides severe syntactic violations. If the early familiarity check identifies the orthographic form of the word, it should be able to respond to orthographic errors (e.g., *posibility*), but not anomalous use of existing morphological forms (e.g., *eats* for *eat*). The model does not answer the question of why some types of errors are detected while others are not, nor the question of why readers do not always notice the same error.

Finally, Warren (2011) argues that the E-Z Reader model will be incomplete without allowing some role for even higher-level influences, based on research on semantic anomalies. Readers sometimes fail to notice semantic anomalies, suggesting that processing is sometimes shallow (Ferreira et al., 2002). “If different readers, reading for different purposes, perform post-lexical processing more or less quickly or completely […], the precise combination of reader, purpose and motivation will affect the patterns of eye movements to semantic violations” (Warren, 2011, p. 922). In our study, we examine how error detection differs between readers with differences in grammatical awareness and proficiency.

### 2.3. The error detection paradigm

Both the eye-tracking and error detection paradigms can be used to measure attention during reading. Here we assume that eye-tracking provides a more sensitive measure than error detection. Yet, the exact correlations between the two measures is not well-explored. It may be the case that the error detection paradigm treats two events as the same, while they involve different eye movements. Although we assume that error detection is more rough, there are several advantages to using this paradigm for our purpose. In the previous eye-tracking studies of ungrammaticality, sentences were presented individually. With error detection, we can introduce participants to long, consecutive texts, simulating natural text reading. Furthermore, we can include many different types of grammar errors, unlike previous eye-tracking studies which have included relatively few error types (e.g., pragmatic vs. syntactic). Having many different types of errors in different conditions would result in a long and tiresome eye-tracking experiment. Finally, using error detection, we can get participants’ feedback on where errors occur, in a fast way, not having to ask after every trial. Although, error detection can only provide a rough measurement for attention during reading, it can provide insights into which types of errors are more noticed than others, and which other factors than error type is likely to play a role. The results are therefore relevant to future eye-tracking studies and processing models. If differences are found using error detection, they are also likely to be found using a presumably more sensitive measure such as eye-tracking.

In our error detection study in Danish, we included one type of syntactic error (*ASV for AVS, see below) and two types of morphological errors (confusion of infinitive and present tense, and gender mismatches between articles or adjectives in NPs), as well as various common orthographic errors. These errors were chosen because they represent a broad range of error types, and they are all attested in natural L1 and/or L2 production, however with different frequencies. For example, ungrammatical verb-third word order (*ASV) instead of grammatical verb-second word order (AVS) is common in L2 Danish (Søby and Kristensen, 2019; Søby and Kristensen, to appear), but rare in L1 Danish, apart from multiethnic urban vernaculars (Quist, 2008). The three types of grammar errors naturally occur in different conditions, varying with respect to error frequency (measured as error rates in L1 production), and/or phonological similarity to the correct form, or placement in the sentence. Since the stimuli is based on naturally occurring errors, error frequency and phonological similarity tend to co-vary.

### 2.4. Attention to errors during reading

Many potential contributing factors besides error type might influence whether a reader reacts to an error. In this section, we elaborate on why some of the factors we are examining in our study are relevant to include, namely error frequency, phonological similarity to the correct form, and, for word order, placement in the sentence. Finally, we elaborate on the potential role of participants’ own production of errors, and individual differences in error perception.

Previous letter detection studies and change-blindness studies review a wide a range of factors which can influence attention during reading (e.g., Smith and Groat, 1979; Sturt et al., 2004; Vinther et al., 2015; Christensen et al., 2021). For example, Smith and Groat (1979) found that the position on the line and in the sentence influenced detection of the letter *e*, so that the outer positions were more prominent than the middle. Using V3 errors with a length manipulation, we examine whether position effects within the sentence are also found for grammar errors.

The main focus of our study is on the role of error frequency. We hypothesize that error frequency, which is tied to the predictability of the error, predicts perception patterns. According to prediction-based approaches to sentence processing, unexpected input attracts attention (Kamide, 2008; Levy, 2008; Christiansen and Chater, 2016). If a reader sees input with common errors, the model will be updated according to the input, meaning that frequent errors should be predicted by the model, and thus should attract less attention than infrequent errors.

Besides error frequency, we expect that phonological similarity to the correct form negatively influences detection rates for grammar errors, in line with Larigauderie et al. (2020) who compared spelling errors which were either phonologically similar to or distinct from the correct form. One example from our stimuli is confusion of homophone verb pairs, such as present tense *kører* and infinitive *køre*, both pronounced [ˈkʰøːɐ]. We expect that confusion of heterophone verb pairs such as *rejser* [ˈʁɑjˀsɐ] and *rejse* [ˈʁɑjsə] will have higher detection rates. When the correct form is homophone to the error, the error is not grammatical in that context, but it is phonologically correct, and may therefore not disturb reading. For such silent errors readers may use all available cues whether they are phonological or orthographic (*cf.* Carassco-Ortiz and Frenck-Mestre, 2014). The E-Z Reader model does not account for homophony effects, but it may predict that the phonological form is more easily identified for homophone compared to heterophone errors in the later stage of lexical processing (*L_2_*). The error frequency and phonological similarity to the correct form tend to co-vary, because L1 speakers of Danish produce more errors when for instance present tense and infinitive forms are homophone. Thus, effects of phonological similarity and frequency are often difficult to disentangle.

On top of that, individual differences are likely to influence error detection. If a type of error is frequent in a person’s production, e.g., omitting the-r on verbs in present tense: *han køre ‘he drive.inf,’ the rules for verbal inflection may not be fully mastered. It therefore seems likely that this person will overlook this type of error in general. Individual differences in the perception of what constitutes an error in a specific situation could also be a factor: How correct or incorrect on a continuum is an error to a specific reader? How do individual readers differentiate between unusual language and outright errors? And is the perception affected by the context in which it is read, e.g., experimental vs. natural? Our study is not equipped to answer these questions. Studies show that tolerance for various errors can be modulated by participants’ perception of the speaker, so that the tolerance and willingness to repair is higher when the speaker is perceived as non-native (Konieczny et al., 1994; Hanulíková et al., 2012; Gibson et al., 2017).

In the public debate and prescriptive literature, some errors are pointed out as typical or basic errors, while other errors are much less debated or accentuated. Publically debated errors may be more prominent to readers (Blom and Ejstrup, 2019b). In Denmark, missing present tense-r is often accentuated in normative discourse. Blom and Ejstrup (2019b) found that readers’ intolerance for errors are modulated by the type of error. Their participants were more annoyed with typical and basic grammar/spelling errors than with atypical and complicated errors. The missing present tense -*r* was the most annoying error. The authors also found a correlation between participants’ irritation (with a specific item) and the number of errors detected, so that the more errors participants detected in general, the more irritated they were with that item.

### 2.5. The current study

The current study examines native speakers’ attendance to different types of syntactic, morphological and orthographic errors (found in L1 and/or L2 Danish) during reading. We asked Danish high school students to read and comprehend two texts, while underlining all errors they noticed. We also tested their basic grammar skills, using a grammar quiz. The study included one type of syntactic error (V3 word order) and two types of morphological errors (confusion of infinitive and present tense, and gender mismatches between articles or adjectives in NPs), as well as various common orthographic errors. V3 errors are the least frequent, and orthographic errors the most common. In a corpus of 71 high school essays, we found 10 V3 errors, 16 gender mismatches in indefinite articles, 51 gender mismatches in adjectives, 178 confusions of infinitive and present tense, and 1,099 orthographic errors.

The study is designed as a four-in-one study. Each error type (V3, verb, NP, orthographic) constitutes its own subexperiment and appears in different conditions, controlled for a number of variables. We cannot directly compare attention to the four types, as there are too many confound variables, such as their position in the sentences and in the text. Thus, we only indirectly compare the detection rates for the three overall error categories (syntactic, morphological, orthographic) using descriptive statistics.

We examine the relationship between the type of errors that young readers tend to overlook in texts, the type of errors these young readers produce themselves (in the grammar quiz), and the type of errors that are typical of their age group in general (based on corpus studies of high school essays). Our expectation is that attention to a specific type of grammar error is not only a matter of the reader’s explicit grammar awareness (as measured in the grammar quiz), but also of whether the specific type of error is common in everyday texts by native speakers. If a specific type is frequent among the peers of the reader, the reader may have more exposure to this type of anomaly and a mental representation of the error, i.e., common errors should be predicted to occur in input, based on prediction theory (Kamide, 2008; Christiansen and Chater, 2016). The reader may therefore find it less striking and be less likely to detect it than errors that are infrequent in texts written by peers, e.g., those found in L2 Danish. This means that for the overall categories of errors (syntactic, morphological and orthographic), we expect that the syntactic errors (V3 errors) have higher detection rates than morphological and orthographic errors, because V3 errors are rare in L1 writing (and are visually large). We also expect readers to overlook orthographic errors the most, because orthographic errors are highly frequent in the L1 writing.

Finally, for the two morphological subtypes of grammar errors (confusion of infinitive and present tense, and gender mismatches between articles or adjectives in NPs), we examine how error frequency and phonological similarity to the correct form may affect attention to errors. For the word order errors, we examine position effects within the sentence. The specific conditions and hypotheses for the three subtypes of grammar errors are presented in the results section where they are treated as three subexperiments. The fourth subexperiment on different types of orthographic errors is primarily included to create variation in the stimuli.

## 3. Methods

### 3.1. Participants

The participants were recruited from three different Danish upper secondary education programs (STX, HTX, and HHX).^2^ Data were collected in August 2019 at six schools located in and around Copenhagen and Roskilde. Two hundred and forty students from 10 classes participated. We excluded participants with dyslexia (18), with late acquisition of Danish (>6 years, Hyltenstam and Abrahamsson, 2003) (2), or participants who misunderstood or did not finish the reading task (9). This left 211 participants in the analysis (98 women, 113 men), 17–20 years of age (M = 18.31 years; SD = 0.67 years). The majority were part of the STX Program (130), followed by HHX (43), and HTX (38). All participants (or their parents) gave informed written consent prior to the experiment. The study was approved by local research ethics committee at University of Copenhagen, and followed GDPR.

### 3.2. Experimental tasks and materials

The experimental tasks consisted of a reading task (section 3.2.1) which was followed by a grammar quiz and a questionnaire (section 3.2.2). All test materials are found in Supplementary material (section 3).

#### 3.2.1. Reading task

The reading task consisted of two texts, A (689–692 words) and B (831–832 words). Every participant read both texts. There were four versions of the reading task material to ensure that each participant only saw the same item in one condition. That is, when reading the same sentence in the text, participants reading version 1 were presented with the verb error in one condition, participants reading version 2 were presented with it in another condition, etc. Each participant was presented with a total of 100 errors in text A and B together. Table 1 shows the distribution on subtypes. To avoid priming effects, target items did not occur elsewhere in the texts.

**Table 1:** Error types, conditions and number of target items in the reading task (text A + B).

Error types		Items
**V3 errors (2 conditions, 8 items per condition)**		**16** ^**a**^
1) After short adverbial:	*ogkl.14 han ankommer til Berlin*and o’clock 2hearrive.prs in Berlin‘and at 2 o’clock, he arrives in Berlin’	8
2) After long adverbial:	*ogførst ud på eftermiddagenhan ankommer til Berlin*and first out on afternoon.def he arrive.prs in Berlin‘and first in the afternoon, he arrives in Berlin’	8
**Verb errors (4 conditions, 8 items per condition)**		**32**
1) Homophone; Present tense for infinitive:	*hanvilkører* [ˈkʰøːɐ]hewill drive.prs‘he’ll drive’	8
2) Homophone; Infinitive for present tense:	*hankøre* [ˈkʰøːɐ]hedrive.inf‘he drives’	8
3) Heterophone; Present tense for infinitive:	*hanvilrejser* [ˈʁɑjˀsɐ]hewill travel .prs ‘he’ll travel’	8
4) Heterophone; Infinitive for present tense:	*hanrejse* [ˈʁɑjsə]hetravel .inf ‘he travels’	8
**NP errors (4 conditions, 8 items per condition)**		**32**
1) Mismatch ADJ + N; Uter for neuter:	*etdejligkæledyr*art.nlovely-upet.n‘a lovely pet’	8
2) Mismatch ADJ + N; Neuter for uter:	*endejlig-tundulat*art.ulovely-nbudgie.u‘a lovely budgie’	8
3) Mismatch ART + N; Uter for neuter:	*endejlig-tkæledyr*art.ulovely-npet.n‘a lovely pet’	8
4) Mismatch ART + N; Neuter for uter:	*etdejligundulat*art.nlovely-ubudgie.u‘a lovely budgie’	8
**Misspellings (4 types — 5 of each type)**		**20** ^**b**^
1) Missing double consonant, e.g., *startskudet* for *startskuddet* ‘the starting signal’		5
2) Split compounds, e.g., *by vandring* for *byvandring ‘*city walk’		5
3) Missing silent letter, e.g., *siste* [ˈsisd̥ə]/[ˈsisd̥] for *sidste* [ˈsisd̥ə]/[ˈsisd̥] ‘last’		5
4) Reduction of syllable, e.g., *virklig* [ˈʋiɐ̯ɡ̊li] for *virkelig* [ˈʋiɐ̯ɡ̊li] ‘really’		5
**Total**		**100**

a The V3 errors in version 1 + 2 were identical. The V3 errors in version 3 + 4 were also identical.

b The 20 spelling errors were identical in all four versions of the reading task.

A further description of the stimuli is presented in the sections on each subexperiment. We varied the order of text A and B, so that half of the participants read A before B, and the other half read B before A. Thus, there were eight versions of the reading task in print.

#### 3.2.2. Questionnaire and grammar quiz

The questionnaire addressed the participants’ language and dialectal background as well as their attitude to language errors. The purpose of the grammar quiz was to ensure that the participants had the basic grammatical prerequisites to notice errors in the reading task. The grammar quiz included tests on all four types of errors, i.e., verb-second word order after sentence-initial adverbials, verb conjugations in infinitive and present tense, conjugation of adjectives, gender of indefinite articles, and spelling of the four types of target words. Most of the tasks were forced-choice between two options.

### 3.3. Procedure

The participants were informed that the study was about speed-reading and what readers notice when skimming a text. In the reading task, their task was to underline language errors. Participants had max. 7 min to read each text (A and B). Participants were instructed to skim as fast as possible and finish reading the whole text so they could answer the comprehension questions. Whenever they noticed a language error, they should underline it, but they should avoid going back in the text. Language errors were defined as different types of spelling and grammar errors, but not punctuation. They were instructed to underline the whole word containing the error, or multiple words if they were in the wrong order. Underlinings could be canceled with a vertical line. Use of dictionaries and online tools were not allowed.

The researcher registered the starting time and gave statuses on remaining time. When the students finished reading the text, they wrote the finishing time and put the text away (if they did not finish, they marked how far in the text they got). The same procedure was repeated for the second text. Finally, the students completed the comprehension questions for both texts, the questionnaire and the grammar quiz. The whole session lasted around 45 min.

## 4. Analysis

The error detection data were analyzed with general linear mixed effects models for binomial data in RStudio (R Core Team, 2022, version 2022.07.1), using the lme4 package (Bates et al., 2015, ver. 1.1.30). *p*-values were obtained using the lmerTest package (Kuznetsova et al., 2017, ver. 3.1.3). The dependent variable for all models was detection, i.e., whether the error was detected (=1) or not (=0). We did not penalize false hits. The conditions for each of the four error types were included in the models as fixed effects (p is the probability of correctly detecting an error):

1. Model for V3 errors: log(p/1-p)^3^ = Adverbial length [short vs. long] + Total grammar score + (1|Participant) + (1|Item) + Residuals
2. Model for Verb errors: log(p/1-p) = Type [infinitive for present tense vs. present tense for infinitive]*Homophony [homophone vs. heterophone pairs] + Total grammar score + (1|Participant) + (1|Item) + Residuals
3. Model for NP errors: log(p/1-p) = Type [agreement with article vs. adjective]*Gender [uter for neuter vs. neuter for uter] + Total grammar score + (1|Participant) + (1|Item) + Residuals
4. Model for orthographic errors: log(p/1-p) = Type [four different] + Spelling score + (1|Participant) + (1|Item) + Residuals

All models included random intercepts for participant and item. All models also included the scores from the grammar quiz. Participants made few wrong answers in the grammar tasks, so we summarized the results from the individual grammar-related tasks and included a total grammar score as a fixed effect in the models for detection of the three types of grammar errors. The model for orthographic errors included the score from the spelling task in the quiz as a fixed effect.

The models for the four error types did not include random slopes, presentation order (i.e., placement in the text) or irritation scores, as the models failed to converge when they were included. Only one subtype, NP errors, showed an uninterpretable effect of presentation order.

The output of the regression model was in logodds space. To increase interpretability, they were converted back to probabilities and plotted. Thus, the plots for the morphological errors show the models’ predicted probabilities of detecting the target.

Finally, we made a general model, collapsing all error subtypes, with accuracy in percentage as the dependent variable, only including irritation scores as a fixed effect (see normal Q-Q plot in Supplementary Figure 3):

1. Model for all errors: accuracy (%) = Irritation score + Residuals.

## 5. General results

The participants detected 54% of all errors in the two texts (Table 2). As expected, the highest detection rate was found for syntactic errors (71% of all items were detected), followed by the two types of morphological errors (55% detected for NP errors; 59% for verb errors), and the lowest rate was found for orthographic errors (33%). The study is not designed to directly compare these overall categories (syntactic, morphological and orthographic), as there are a number of confounds, such as their position in the sentences and in the text. We therefore do not conduct any statistical tests between them. More detailed results are presented in the sections on each of the four error types (subexperiments).

### 5.1. Individual variation

**Figure 1:**
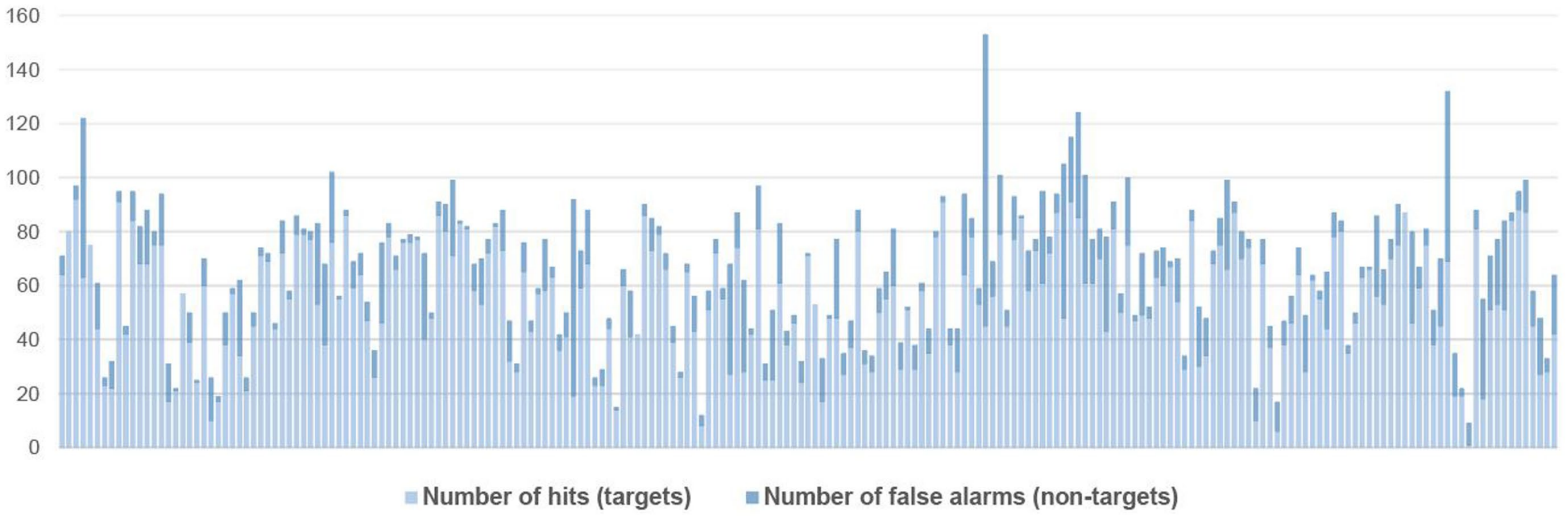
Number of underlinings (hits and false alarms) per participant.

As seen in Figure 1, there was individual variation among the participants, with respect to the number of words they underlined, and the share of correct (hits) vs. incorrect underlinings (false alarms). Out of 321,145 words, participants underlined 18,041 words (M = 85,50 words, SD = 31,38 words, range = 9–227 words). Of these only 2,565 words were not part of a target, i.e., false alarms (M = 12,16 words, SD = 13,59 words, range = 0–108). In total, 11,490 targets were underlined, i.e., hits (M = 54,45 words, SD = 21,32 words, range = 1–92 words). Notice that a target can consist of several words (targets are defined in the sections on the subexperiments).

In principle, participants could underline all words in the text and thus detect all errors, resulting in the highest possible score. This, however, was not an issue in general as participants only underlined 0.8% non-target words in the texts (2,565 out of 321,145 words). Figure 1 shows that most participants were relatively exact in their underlinings, apart from 10 participants who had more false alarms than hits.

In the grammar quiz, participants generally made few errors (see sections on subexperiments). In the three grammar tasks (word order, NP agreement and verb conjugations), the highest possible score was 17, one point for each correct answer. Participants’ scores had an average of 16.76 (SD = 0.67, range: 11–17). The Supplementary material (section 1.2) include a plot of the total quiz scores (grammar and spelling tasks) and the number of detected errors per participants.

**Table 2:** Number of errors in texts and share of detected errors.

Category type	Errors in texts (*N*)	Detected targets (*N*)	Share of detected targets (%)
Syntax			
V3	3,376	2,398	71.03%
Morphology			
Verb errors	6,752	3,992	59.12%
NP errors	6,752	3,719	55.08%
Orthography			
Misspellings	4,220	1,381	32.73%
**Total**	**21,100**	**11,490**	**54.45%**

The general model of all error types (5) included the participants’ irritation scores (*cf.* Supplementary Table 13). We found a small effect of irritation ( $\hat{\beta }$ = 1.82, SE = 0.40, *t* = 4.57, *p* < 0.001), so that the more annoyed participants state to be with language errors, the more errors they detected in the reading task (see plots in Supplementary material, section 1.2).

## 6. Subexperiments

In the following sections, we present the hypotheses, stimuli and results for each of the four subtypes of errors. Sections 6.1–6.3 describe the three subexperiments on grammar errors. Section 6.4 describes the subexperiment on orthographic errors. The Supplementary material show all stimuli (section 2) and model results for the orthographic errors (section 1.1).

For the grammar errors, we start each section with information on error frequencies in L1 production. The error frequencies are based on a corpus of 71 high school essays from a final exam (127,957 words; 71 participants). For the morphological errors, we calculated the error rate by dividing the number of incorrect tokens with the number of correct and incorrect tokens. As an example, when a reader sees a verb in present tense, the error rate reflects how often the verb is incorrect. For the orthographic errors, the error rate is calculated by dividing the number of errors with the number of words in the corpus. For the syntactic errors, we report the absolute number of errors. Since there was a limited number of tokens for certain types of errors, we only use descriptive statistics (not inferential statistics) when accessing differences in error frequency.

### 6.1. V3 errors

A common word order error in L2 Danish is placing the verb in third position (V3), instead of second (V2; Søby and Kristensen, to appear). In (1a), the adverbial *nu* ‘now’ is placed in first position, followed by the subject *jeg* ‘I’ in second position, and the verb *bor* ‘live’ in third position. In the corrected version of the sentence in (1b), the verb is correctly placed in second position (the mandatory position for finite verbs in Danish main clauses).

**Table:** 

(1)	a. [original]	*Men nujeg bor iDenmark
		‘butnow Ilive in Denmark’
	b. [corrected]	*Men nubor jeg iDanmark*
		‘butnow live Iin Denmark’

In the L1 corpus of high school essays, we only found 10 V3 errors. V3 errors are generally not considered typical L1 errors, but may occur in informal texts written by speakers of multiethnic urban vernaculars (Quist, 2008).

We expected these errors to be highly noticed by native speakers for two reasons. First, they are rare in L1 production. Second, large elements, i.e., entire words, are misplaced. In the experiment, the V3 errors were either presented after a short sentence-initial adverbial (1–2 words, consisting of 5–12 characters including spaces) or a long adverbial (4–6 words, 26–39 characters). In L2 Danish, V3 word order most frequently occurs after adverbials, both short and long (Søby and Kristensen, to appear). Examples of the stimuli are shown in Table 3. Previous letter detection studies have found position effects, so that elements in the start or end of a sentence tend to be more prominent than in the middle (Smith and Groat, 1979). We therefore examined whether participants would detect more V3 errors after a short adverbial than a long adverbial.

**Table 3:** Conditions, number of V3 errors in texts and share of detected errors.

Conditions	Errors in texts (*N*)	Detected targets (*N*)	Share of detected targets (%)
**Short A***ogkl.14 han ankommer til Berlin* and o’clock 2hearrive.prsin Berlin ‘and at 2 o’clock, he arrives in Berlin’	1,688	1,200	**71.09%**
**Long A***ogførstud på eftermiddagen han ankommer til Berlin* and first out on afternoon.def hearrive.prsin Berlin ‘and first in the afternoon, he arrives in Berlin’	1,688	1,198	**70.97%**

The target verbs were all in present or perfect tense, and subjects were either pronouns, proper names or nouns in the definite form, with varying lengths. The texts also included 16 similar correct constructions with AVS, i.e., V2 word order (8 after short adverbials; 8 after long). All stimuli can be seen in Supplementary material (section 2).

The V3 errors were considered detected when either the adverbial, subject or verb was underlined by a participant, since the order of subject and verb would be correct if the adverbial was placed elsewhere. In Table 3, the number and share of detected targets are seen. There were no effects of adverbial length ( $\hat{\beta }$ = −0.03, SE = 0.09, *z* = −0.38, *p* = 0.70), but there was an effect of total grammar score ( $\hat{\beta }$ = 0.73, SE = 0.16, *z* = 4.51, *p* < 0.001; *cf.* Table 4). The higher grammar score in the quiz, the more V3 errors were detected. In the grammar quiz, participants had to place words in the correct order after conjunctions and adverbials. Out of 633 answers, only 3 were wrong (0.5%), confirming that V3 is not a typical L1 error.

**Table 4:** Model (1) estimates for V3 errors.

Random effects	Variance	Std. dev.		
Participant (intercept)	1.7076	1.3068		
Item (intercept)	0.4177	0.6463		
Fixed effects	Estimate	Std. error	*z*-value	*p*-value
(Intercept)	−10.91748	2.70189	−4.041	5.33e-05^***^
Length	−0.03394	0.08865	−0.383	0.702
Total grammar score (quiz)	0.72652	0.16094	4.514	6.36e-06^***^

Dependent variable: detection (1 = error detected, 0 = error not detected). Significance code: ***p < 0.001.

### 6.2. Verb errors

Confusion of finite and infinite verb forms is the most frequent morphological error in the L1 corpus. More specifically, there are 181 cases of confusion of infinitive and present tense in the L1 corpus. When examining these, the error frequency seems influenced by phonological similarity (Table 5). L1 speakers produce more errors when the two verb forms are homophone (e.g., infinitive *køre* [ˈkʰøːɐ] and present tense *kører* [ˈkʰøːɐ]) than when the verb forms are heterophone (e.g., infinitive *rejse* [ˈʁɑjsə] and present tense *rejser* [ˈʁɑjˀsɐ]). This is both the case when examining the total number of errors and the error rates. For example, the error rate for using infinitive for present tense (homophone verb pairs) is 25%, i.e., out of all correct verbs in present tense (with the same pronunciation in infinitive) plus the cases where infinitive is used for a homophone present tense form, 25% are erroneous. L1 speakers also produce more errors of the type infinitive for present tense (132) than present tense for infinitive (49), i.e., they leave out an -*r* in writing. However, the error rates for the two types of confusion are both 1%, because there are more verbs in present tense in the corpus.

**Table 5:** Error rates in L1 texts, confusion of present tense and infinitive (*N* = 194).

Type and error rates	Homophone	Heterophone
	e.g., *køre(r)* [ˈkʰøːɐ]	e.g., *rejse* [ˈʁɑjsə], *rejser* [ˈʁɑjˀsɐ]
**Target form: present tense**1% errors (12,764 correct present tense verbs^1^)	25%(*N* = 96)	0.30%(*N* = 35)
**Target form: infinitive**1% errors (4,689 correct infinitives^1^)	8.60%(*N* = 37)	1.10%(*N* = 10)

1 Found using an automatic POS tagger [Centre for Language Technology, University of Copenhagen (CST), 2022], manually tagged for homophony.

Based on error rates (which are entangled with phonological similarity), we expected that participants would detect more errors in the heterophone than homophone conditions. We did not expect differences between the two types of target forms (whether the target was infinitive or present tense), as there was no difference in error rates. Finally, the error rates in Table 5 also show a larger difference between the homophone and heterophone conditions when the target is present tense, compared to when the target form is infinitive. This predicts an interaction between homophony and type.

Table 6 shows the four experimental conditions for the verb errors. We used a 2 (heterophone vs. homophone) × 2 (target infinitive vs. present tense) design. Notice, that there is a visual difference between the two types of errors, because in one condition (present tense for infinitive), an extra *-r* is added, while an -*r* is missing in the other condition (infinitive for present tense). The heterophone vs. homophone verb pairs were controlled for length (number of letters in infinitive) and frequency. *T*-tests (correlated samples) showed no significant differences in length or frequency [Det Danske Sprog- og Litteraturselskab (DSL), 2022] for the homophone vs. heterophone verbs. The texts also included a minimum of 32 correct verbs (other lexemes), 8 in each condition. All stimuli can be seen in the Supplementary material (section 2).

**Table 6:** Conditions, number of verb errors in texts and share of detected errors.

Conditions	Errors in texts (*N*)	Detected targets (*N*)	Share of detected targets (%)
**heterophone pairs**	**3,376**	**2,306**	**68.31%**
infinitive for present tense:*han rejse* [ˈʁɑjsə]he travel .inf	1,688	1,178	69.79%
present tense for infinitive:*han vil rejser* [ˈʁɑjˀsɐ]he will travel .prs	1,688	1,128	66.82%
**homophone pairs**	**3,376**	**1,686**	**49.94%**
infinitive for present tense:*han køre* [ˈkʰøːɐ]he drive.inf	1,688	867	51.36%
present tense for infinitive:*han vil kører* [ˈkʰøːɐ]he will drive.prs	1,688	819	48.52%
**Total**	**6,752**	**3,992**	**59.12%**

Table 6 also shows the number and share of detected targets. In the condition present tense for infinitive, a target is considered detected if either the modal and/or the main verb is underlined.

As expected (based on error rates and phonological similarity), we found an effect of homophony ( $\hat{\beta }$ = −1.21, SE = 0.09, *z* = −13.38, *p* < 0.001), so that participants detected more errors in heterophone than homophone pairs. Counter to the expectation based on error rates, we found an effect of type, so that more errors of the type infinitive for present tense were found, than for present tense for infinitive ( $\hat{\beta }$ = −0.20, SE = 0.09, *z* = −2.23, *p* < 0.05). There was no interaction, contrary to the predictions based on error rates (*cf.* Table 7).

**Table 7:** Model (2) estimates for verb errors.

Random effects	Variance	Std. dev.		
Participant (intercept)	2.7768	1.6664		
Item (intercept)	0.2204	0.4695		
Fixed effects	Estimate	Std. error	*z*-value	*p*-value
(Intercept)	−10.75433	2.99431	−3.592	0.000329^***^
Homophony	−1.20594	0.09014	−13.378	<2e-16^***^
Type	−0.20145	0.09025	−2.232	0.025614^*^
Homophony*type (Interaction)	0.01925	0.12440	0.155	0.877033
Total grammar score (quiz)	0.71993	0.17850	4.033	5.5e-05^***^

Dependent variable: detection (1 = error detected, 0 = error not detected). Significance codes: ***p < 0.001, *p < 0.05.

**Figure 2:**
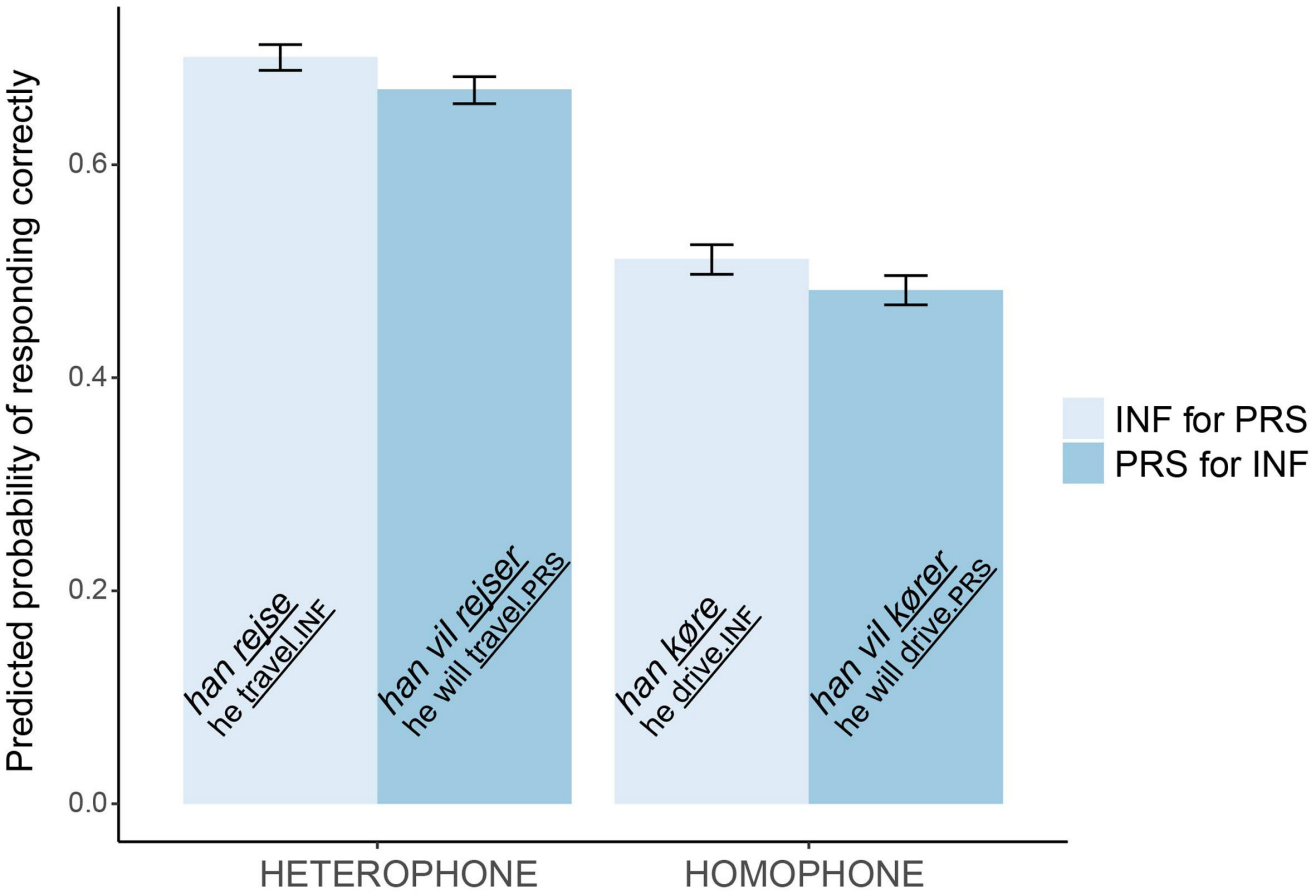
The model’s predicted probabilities of detecting verb errors. Error bars show SDs.

Figure 2 shows the model’s predicted probability of responding correctly (i.e., detecting the error) in the different conditions. The probability of a correct answer (a detected error) is much higher in the heterophone than homophone conditions. Although, the effect of type was significant, the plot shows that it is small. Also, according to the predictions based on error rates, the column with *han køre* should have been the smallest.

Finally, we found an effect of total grammar score ( $\hat{\beta }$ = 0.72, SE = 0.18, *z* = 4.03, *p* < 0.001), so that the higher total grammar score in the quiz, the more verb errors were detected. The grammar quiz contained 8 sentences where participants made a forced choice between infinitive or present tense for a missing verb. Out of 1.688 answers, there were only 25 errors (1.5%), made by 16 students. Twenty-two of 25 errors were in homophone verb pairs, supporting the role of phonological similarity on error production.

### 6.3. NP errors

In Danish, nouns are either uter (most common) or neuter gender. There are two indefinite articles, *en* (uter) and *et* (neuter) ‘a.’ Adjectives are inflected for gender, definiteness, and number. Typically, the suffix -*t ‘*neuter,’ -*e ‘*definite,’ or -*e ‘*plural,’ can be added to the uninflected basic form, corresponding to singular, indefinite, uter gender (Becker-Christensen, 2010). The most common adjective error in the L1 corpus is to leave out a suffix (-*t* or -*e*). Table 8 shows error rates for gender mismatches in adjectives and indefinite articles. Confusing the two indefinite articles is less common than missing gender agreement in adjectives, as seen in the error rates. Using uter for neuter is slightly more common than using neuter for uter.

**Table 8:** Error rates in L1 texts, gender mismatch between indefinite articles or adjectives with noun.

	*N* errors	N correct	Error rate (%)
**Indefinite articles**	**16**	**3,132**	**0.51%**
Uter for neuter (*en* for *et*)	6	984^1^	0.61%
Neuter for uter (*et* for *en*)	10	2,178	0.46%
**Adjectives**	**51**	**2798** ^**2**^	**1.79%**
Uter for neuter (Ø for -*t*)	29	1,368	2.08%
Neuter for uter (-*t* for Ø)	22	1,430	1.49%

1 Number of correct occurrences of et ‘a’ (neuter), found with a POS tagger [Centre for Language Technology, University of Copenhagen (CST), 2022].

2 The number of correct adjectives with a correct -Ø or -*t* suffix. Found with a POS tagger [Centre for Language Technology, University of Copenhagen (CST), 2022]. Manually, the following were removed: adjectives with no/optional gender conjugations (ending with *-sk*, *-vis*), indeclinable adjectives (e.g., *ekstra* ‘extra’), and adjectives ending with a -*t* (e.g., *stolt* ‘proud’).

Based on the error rates, we expected higher detection rates for mismatching articles than for mismatching adjectives, and higher detection rates for neuter for uter more than uter for neuter. The error rates in Table 8 show a slightly larger gender difference for adjectives than for articles, and we therefore predicted an interaction between word class and gender.

The four experimental conditions for the NP errors are seen in Table 9 (2 × 2 design). In continuous speech, there is phonological similarity between the correct and incorrect form in the condition mismatch with adjective, uter for neuter (where the suffix is missing). Notice, that there are also visual differences between the two word class conditions: when manipulating the adjectives, an element (-*t*) is either added or left out. When manipulating the articles, a *t* or an *n* is replaced with each other.

**Table 9:** Number of NP errors in texts and share of detected errors.

Conditions	Errors in texts (*N*)	Detected targets (*N*)	Share of detected targets (%)
**mismatch art + n**	**3,376**	**2034**	**60.25%**
Neuter for uter: *et**dejligundulat*art.nlovely-ubudgie.u‘a lovely budgie’	1,688	1,021	60.49%
Uter for neuter: *en**dejlig-tkæledyr*art.ulovely-npet.n‘a lovely pet’	1,688	1,013	60.01%
**mismatch adj + n**	**3,376**	**1,685**	**49.91%**
Neuter for uter: *en**dejlig-t**undulat*art.ulovely-nbudgie.u‘a lovely budgie’	1,688	959	56.81%
Uter for neuter: *et**dejlig**kæledyr*art.nlovely-upet.n‘a lovely pet’	1,688	726	43.01%
**Total**	**6,752**	**3,719**	**55.08%**

The neuter and uter nouns were controlled for length and frequency. The target items did not have the same syntactic function (e.g., object, subject complement or part of an adverbial) and thus were not in the same position in the sentences. The text also contained a minimum of 32 control items (16 uter NPs; 16 neuter NPs), which were inflected adjectives not already used as targets.

Table 9 shows the number and share of detected targets. Targets were considered detected if min. one of the three words in the NP was underlined.

**Figure 3:**
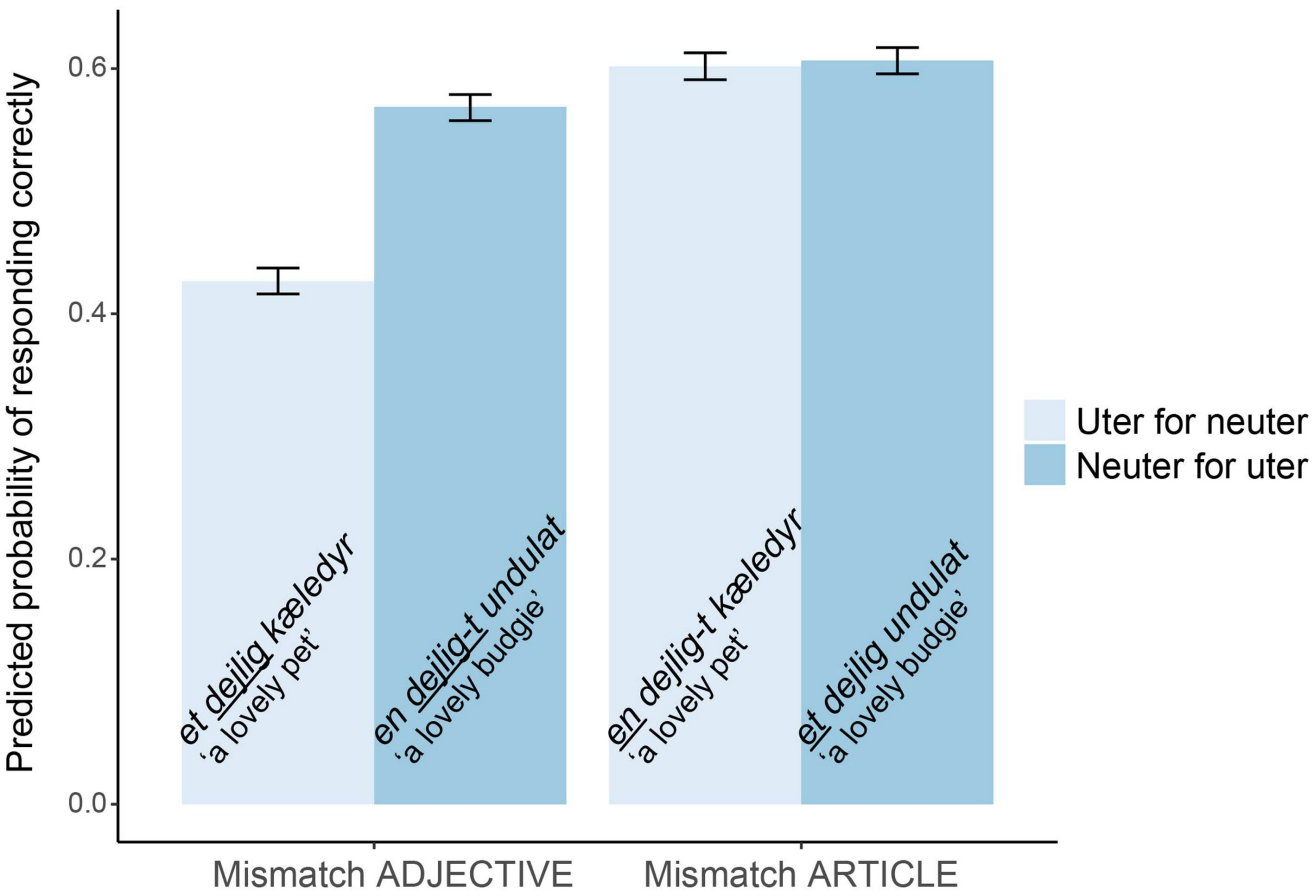
The model’s predicted probabilities of detecting NP errors. Error bars show SDs.

As predicted based on error rates, we found an effect of word class ( $\hat{\beta }$ = 0.90, SE = 0.08, *z* = 11.30, *p* < 0.001), so that mismatches with articles were detected more than mismatches with adjectives. As expected based on error rates, we found an effect of gender ( $\hat{\beta }$ = 0.72, SE = 0.08, *z* = 9.08, *p* < 0.001), so that participants detected more neuter for uter than uter for neuter in general (*cf.* Table 10). We also found the expected interaction ( $\hat{\beta }$ = −0.70, SE = 0.11, *z* = −6.23, *p* < 0.001), which can be seen in Figure 3. It shows the model’s predicted probability of responding correctly (detecting the error) in the different conditions. For the articles, the effect of gender is less pronounced than for the adjectives. The lowest detection rates were found for *et dejlig kæledyr* (mismatch with adjective; uter for neuter), as expected. However, the interaction might also be explained by the phonological similarity to the correct form in this condition, or visual differences between conditions. Perhaps, it is harder to spot a missing -*t* than an extra -*t* or to spot a *t* which is replaced with an *n*. Finally, we found an effect of total grammar score ( $\hat{\beta }$ = 0.42, SE = 0.12, *z* = 3.38, *p* < 0.001), so that the higher total grammar score in the quiz, the more NP errors were detected. In the grammar quiz, participants were given an adjective and asked to insert it before both an uter and a neuter noun. The article task was forced choice, and participants had to choose between uter or neuter indefinite articles for four nouns. There were only 6 errors for the 844 articles (0.7%) and no errors for the 422 adjectives.

**Table 10:** Model (3) estimates for NP errors.

Random effects	Variance	Std. dev.		
Participant (intercept)	1.260	1.1226		
Item (intercept)	0.192	0.4381		
Fixed effects	Estimate	Std. error	*z*-value	*p-*value
(Intercept)	−7.37728	2.07508	−3.555	0.000378^***^
Word class	0.90480	0.08013	11.292	<2e-16^***^
Gender	0.72108	0.07945	9.076	<2e-16^***^
Word class*Gender (interaction)	−0.70256	0.11276	−6.231	4.64e-10^***^
Total grammar score (quiz)	0.41706	0.12357	3.375	0.000738^***^

Dependent variable: detection (1 = error detected, 0 = error not detected). Significance code: ***p < 0.001.

### 6.4. Orthographic errors

In general, we expected common types of misspellings to be noticed less than syntactic and morphological errors. In the high school corpus, orthographic errors are the most common type of error (0.86% of all words are misspelled). The 20 target items were created based on four types of misspellings which others have found to be common in L1 writing (e.g., Blom et al., 2017). Examples can be seen in Table 11. Table 11 also shows the number and shares of detected errors. Most of the errors are phonologically similar to the correct form. Some are entirely homophone (e.g., the error *virklig*), while other errors could be prosodically different, e.g., with respect to vowel length or stress.

**Table 11:** Types of orthographic errors, number of errors in texts and share of detected errors.

Types of orthographic errors (four types — five of each type)	Errors in texts (*N*)	Detected targets (*N*)	Share of detected targets (%)
**Missing double consonant**, e.g., *startskudet* for *startskuddet ‘*the starting signal’	1,055	224	21.23%
**Split compounds**, e.g., *by vandring* for *byvandring* ‘city walk’	1,055	342	32.42%
**Missing silent letter**, e.g., *siste* [ˈsisd̥ə]/[ˈsisd̥] for *sidste* [ˈsisd̥ə]/[ˈsisd̥] ‘last’	1,055	359	34.03%
**Reduction of syllable**, e.g., *virklig* [ˈʋiɐ̯ɡ̊li] for *virkelig* [ˈʋiɐ̯ɡ̊li] ‘really’	1,055	456	43.22%
**Total**	**4,220**	**1,381**	**32.73%**

The only significant effect of type was that reduced syllables were detected more often than missing double consonants, which were noticed the least ( $\hat{\beta }$ = 1.40, SE = 0.55, *z* = 2.56, *p* < 0.05). Finally, there was a significant effect of the score in the spelling task in the quiz, so that the more correct answers participants had in the spelling task, the more orthographic errors participants found in the reading task ( $\hat{\beta }$ = 0.50, SE = 0.08, *z* = 6.52, *p* < 0.001). In the spelling task, participants had to determine whether 8 words were spelled correctly. If not, they should write the correct form. There were 196 errors out of 1.688 answers (12% errors), made by 115 participants (1–5 errors per participant).

## 7. Discussion

Section 7.1 is a summary and discussion of the general findings of the study. In section 7.2, we discuss the relation between error detection rates and two seemingly dominant (and co-varying) factors in our study: the frequency of the error and its phonological similarity to the correct form. Section 7.3 discusses challenges for current and future models of eye movement control in reading and presents our recommendations based on the study.

### 7.1. General findings and effects of explicit grammar awareness

The present study examined the relationship between the type of errors young readers tend to overlook in texts, the type of errors these young readers produce themselves in the grammar quiz, and the type of errors that are typical of their age group in general (based on corpus error rates). When examining attention to naturally occurring grammar anomalies, some factors co-vary. Still, to use ecological stimuli is necessary if future models of language processing are to be able to accommodate naturally occurring, non-standard grammar.

In our study, grammar errors seem to attract more attention than orthographic errors. This finding is in line with Larigauderie et al. (2020) who studied attention to grammatical and orthographic errors in French. Their grammar errors were comparable to ours, as they related to number and gender agreement and misuse of the past participle form in French. Their orthographic errors (like most of ours) did not affect the phonology of the word. Previous proofreading studies of English (Hacker et al., 1994; Shafto, 2015), however, found the opposite pattern, as orthographic errors attracted more attention than grammar errors in their studies. It is likely that this discrepancy stems from differences in what is understood by a grammar error vs. an orthographical error. In Shafto (2015), the grammar errors were heterogeneous ranging from errors in verb agreement and number agreement to punctuation and capitalization errors, thus grouping types of errors which are quite distinct. The orthographic errors also included typos such as letter switches which resulted in an incorrect phonological form, and which are therefore also qualitatively different from the orthographic errors in our study. Larigauderie et al. (2020) found that typos were the most frequently detected type of error. In Hacker et al. (1994), the error categories were not clearly defined. Their grammar errors included errors in verb agreement as well as confusion of word classes (e.g., *affects* for *effects*). Altogether, these differences in the definitions of grammar vs. orthography may explain the seemingly contradictory results.

Error detection is not entirely explained by explicit grammar awareness. In the grammar quiz, the general performance was almost at ceiling with error rates ranging from 0.5% to 1.5% per task. Yet, all readers overlooked errors in the proofreading study.

Although there were generally few errors in the responses to the grammar quiz, the participants’ total score in the grammar quiz did explain some of the variance in the detection rates. For the three types of grammar errors (V3 word order, verb errors, NP errors), we found an effect of the total grammar score, so that the more correct answers participants had in the three grammar tasks in the quiz, the more errors they detected. Similarly, the more correct answers participants had in the spelling task, the more orthographic errors they detected. Finally, we found that the more annoyed with language errors participants reported to be, the more errors they detected.

Unlike most previous psycholinguistic studies which either group many different types of grammar errors into one experimental condition (Hacker et al., 1994; Shafto, 2015) or only investigate one specific type as representative of all grammar errors (often using the cover term *syntactic violations*), our study distinguishes between different types of grammar errors. The descriptive statistics showed differences in detection rates between syntactic and morphological errors in our study, which seems to suggest that not all grammar errors are treated alike. Future eye-tracking studies may determine if this pattern is not just due to quantitative differences (degree of attention), but also due to qualitative differences (differences in how they are processed and attended to).

### 7.2. The relation between what students typically produce and what they notice

Models of natural reading processing must deal with naturally occurring errors. Yet, a complication of using naturally occurring errors is that several factors co-vary between conditions. In the following sections, we discuss two main potential contributing factors when it comes to readers’ perception of and attention to grammar errors in Danish: the frequency of the error (section 7.2.1) and the phonological similarity between the error and the correct form (section 7.2.2).

#### 7.2.1. Error frequency

Our study suggests that the frequency of grammar errors is a relevant factor to include in future models of eye movements during reading. Attention to a specific type of grammar error is not only a matter of the reader’s explicit grammar awareness (as measured in the grammar quiz). If a specific type is frequent among the peers of the reader, the reader may have more exposure to this type of error and a mental representation of it. The reader may therefore find it less striking and be less likely to detect it compared to errors that are infrequent in texts written by peers. According to the descriptive statistics in our study, the error detection rates for the three overall error categories (syntactic > morphological > orthographic) were inversely proportional with the error rates in L1 writing. Syntactic errors have the lowest error rates in L1 writing and the highest detection rates. Orthographic errors have the highest error rates and the lowest detection rates. Within the three grammar subexperiments, we also found that error types with relatively high error rates (errors in homophone verb pairs, mismatching adjectives in NPs, overuse of uter in NPs) had lower detection rates than errors with lower error rates (errors in heterophone verb pairs, mismatching articles in NPs, overuse of neuter in NPs).

Yet, frequency is not the only possible explanation to these results. The higher share of detected syntactic errors could be influenced by differences in size (manipulating word order vs. letters). The homophony effect for verb errors is closely tied to the phonological similarity to the correct form (section 7.2.2.). In the subexperiment on NPs, phonological similarity to the correct form may also explain the interaction between word class and gender (section 7.2.2). Furthermore, frequency and word class co-varied. Also, the effect of word class could be influenced by differences in the placement of the error within the NP. It may be that phrase-initial errors (such as the article errors) attract more attention than errors placed in the middle of a phrase (such as the adjective errors). Thus, future studies are needed, in which effects of position in the phrase and frequency can be distinguished — and if possible, in which effects of frequency can be distinguished from phonological similarity to the correct form.

These reservations aside, it seems likely that frequency plays an important part in error detection, and that the role of frequency is worth studying in future studies with more controlled and less confounded stimuli. Frequency is, as mentioned in the introduction, tied to predictability. According to prediction-based approaches to sentence processing, unexpected input attracts attention (Kamide, 2008; Levy, 2008; Christiansen and Chater, 2016). If a reader sees input with frequent errors, the model will be updated according to the input, meaning that frequent errors should be predicted by the model, and thus should attract less attention than infrequent errors. The error rates in our study were based on texts written by high school students. We do not assume that high school students read each other’s essays, but the errors they produce in school essays are likely to occur in their writing in general, including informal text directed at their peers. Furthermore, we assume that the error production patterns found in high school texts to a large extent reflect the error types found in the media.

Frequency does not explain all findings and it seems to be interacting with other factors in our study. Not all predictions based on error rates were confirmed: we did not expect an effect of type for the verb errors, but found higher detection rates for infinitive for present tense than vice versa. In the public debate and prescriptive literature, missing present tense -*r* is often accentuated as a typical or basic error (Blom and Ejstrup, 2019b), and in the study by Blom and Ejstrup (2019a), participants rated the missing present tense -*r* as the most annoying error of all included errors. This special status of the missing *-r* in present tense might explain why this error type was noticed more than the superfluous -*r* on infinitives, although the frequency in production (as measured by error rates) does not differ between the two. If looking at occurrences per 1,000 words, omitting the -r is, in fact, more frequent in written texts. Counter to our expectations, we did not find an interaction between homophony and type. The surprising result might also be explained by the great prescriptive focus on the most frequent error type (homophone; infinitive for present tense).

In our study, frequency measures were based on error rates in a small corpus of naturally occurring L1 texts. For erroneous use of gender in articles, the error rates were based on only 16 article errors, and the distribution between uter and neuter gender in errors may well be different in a larger corpus. Future studies with a larger corpus may use inferential statistics for a more adequate calculation and assessment of differences in error rates. They may also consider the pros and cons of using error rates vs. raw frequency (errors per 1,000 running words) as the basic measure. In most cases, these measures lead to the same predictions, but in one case, type for verb errors, our frequency-based predictions would have been different if we had based them on occurrences per 1,000 running words, instead of error rates. Homophony set aside, there are more errors per 1,000 words where the target form is present tense (1.02) than when it is infinitive (0.37). Thus, infinitive for present tense should be least noticed. This was, however, not the case, and this frequency measurement therefore does not seem better at predicting error detection than error rates.

To conclude, frequency (measured by error rates) in most cases predicted detection rates of different types of errors. Due to the confounded nature of the highly ecological error types in the stimuli, we cannot determine the exact nature of the interplay with other contributing factors.

#### 7.2.2. Phonological similarity to the correct form

In naturally occurring language we often find errors that intersect grammar and phonology. Since we aimed to study error detection of naturally occurring grammar errors, our stimuli included such intersectional errors. We contrasted grammar errors where the confused forms were phonologically identical (homophone) with errors where the two forms were clearly distinct in pronunciation (heterophone). Our study showed significantly lower detection rates for verb errors in the homophone condition compared to the heterophone condition. These results suggest that phonology interferes with grammatical processing during error detection. Yet, the difference between homophone and heterophone forms may also be due to differences in frequency, as error rates in L1 writing are higher when the present tense and infinitive are homophone. In the verb error subexperiment, we therefore cannot disentangle the effect of phonological interference from that of frequency. Still, we find it plausible that phonological interference constitutes a separate effect when taking into account the findings from the subexperiment on NP errors. For NP errors, detection rates were low when the adjective was inflected in uter instead of the correct neuter form (e.g., *dejlig* instead of *dejligt*). This error with a missing -*t* is not only visually similar to the correct form (*cf.* section 7.3), but also phonologically similar. In distinct speech the final [d̥] in *dejligt* may be pronounced, but in running speech there is usually no audible difference. This similarity between forms may explain why we found an interaction between gender and word class. Frequency differences in error rates may also account for this effect. Yet, the differences in frequency are small. It therefore seems more likely that phonological similarity plays a key role in explaining the low detection rates for uter for neuter in adjectives.

Errors that intersect the boundary between grammar and phonology are not unique to Danish. “Silent suffix” errors with confusion of homophone verb forms are also frequent in other languages. In Dutch the 1st person verb *word* and the 3rd person verb *wordt* have the same pronunciation and are commonly confused (Sandra et al., 2004). In French, there is no audible difference between the verb forms *mange*, *manges* and *mangent*, and ERP studies show that responses to confusion of such homophone verb forms differs from responses to confusion of heterophone verb forms like *mange* vs. *mangez* (Carassco-Ortiz and Frenck-Mestre, 2014). This finding is in line with Larigauderie et al. (2020) who found that typographical errors (i.e., incorrect successions of letters resulting in incorrect phonology) are more frequently detected than orthographic errors which did not affect the phonology of the word. Potential interference from phonology is not limited to confusion of verb forms. The confusion of English *its* and *it’s* is a prime example. Although our study cannot disentangle effects of phonological similarity from error frequency, we recommend that future eye-tracking models of reading and sentence processing models in general consider the possible role of phonological resemblance of errors to correct forms.

### 7.3. Challenges for current and future models of eye movement control in reading

Presumably, the error detection measure is less sensitive than eye-tracking. Although the degree of correlation between the two measures is uncertain, we assume that the overall results could be replicated using eye-tracking, which is a natural next step. More fine-grained differences may also be detected using eye-tracking, e.g., it may be that eye movements are affected, though errors are not underlined by the participant. This was, however, not found in the eye-tracking study by Huang and Staub (2021). Disruption in eye movement measures caused by transposition errors were only found in those sentences participants judged to be ungrammatical. The majority of previous eye-tracking studies of ungrammaticality did not ask participants whether they noticed and perceived the individual errors as ungrammatical or not. Using the error detection paradigm, we collected this information without interrupting participants’ reading excessively and found that attention to different types of naturally occurring errors is not uniform. This variation in the reader’s attention and response to errors poses a challenge to the major present models of eye movement control in reading (Reichle et al., 2003; Engbert et al., 2005). The E-Z Reader model (Reichle et al., 2009) addresses reactions to severe syntactic violations, but does not address what happens when readers encounter misspellings or other types of grammar errors. Results from previous eye-tracking studies of ungrammaticality indicate that different types of grammar errors (e.g., V3 and morphological agreement errors) elicit similar responses in participants’ eye movements across languages, with similar time courses (*cf.* section 2.1) — including the very early effects, which E-Z Reader explicitly predicts for syntactic violations. If attention to different types of errors should be integrated in the E-Z Reader model, a first step could be to integrate detection of orthographic errors as part of the early familiarity check, and to account for both morphological and syntactic errors.

The E-Z Reader model does not explain why some errors are detected while others go by unnoticed, and why different readers do not always notice the same error. Also, as Warren (2011) points out, the model does not consider the precise combination of reader and the purpose or motivation for the reading. Our study both shows an effect of participants’ explicit grammar awareness and general irritation with errors on detection rates.

In our study, we have demonstrated the complexity of measuring error frequency and determining when there is phonological similarity. It is therefore challenging to integrate these factors in models of eye movement control during reading. Still, the two factors are entangled, and even a rough measure of error frequency would improve current and future models when dealing with reading of everyday texts.

Previous letter detection experiments (Smith and Groat, 1979) have found position effects, e.g., that elements in the start or end of a line or within a sentence tend to be more prominent than elements in the middle. Our study on V3 errors manipulated the length of the sentence-initial adverbial, but we found no effects of the placement in the sentence (close to the start vs. further toward the middle). This lack of an effect of position was confirmed in an eye-tracking study where Norwegian readers read similar types of V3 with long and short adverbials (Søby et al., 2023). Smith and Groat (1979) did not consider different sentence structures in their analysis, only numerical order of the words, and the position effects varied between items. Further studies are needed to test the potential role of error position within the sentence.

For the verb and NP errors, there were visual differences between elements that were deleted, added and replaced with other elements. The NP data suggest that replacing two elements with another (i.e., *-t* and *-n* in indefinite articles) is noticed more than when an element is added or missing (-*t* in adjectives). However, for verb errors, a missing -*r* was more noticed than an extra -*r*. It therefore seems that other factors than visual differences are more important, e.g., word class or error frequency.

In this study, we have examined outright errors which both deviated from the norms defined by the Danish Language Council and from most participants’ own answers in the grammar quiz. Language norms, however, are subject to language change and sociolinguistic variation. Natural texts therefore both contain outright errors and language anomalies in the gray zone between language errors and language variation. For instance, the inflection of Danish modal verbs seem to be subject to language change. In written production most high school students do not inflect the Danish modal verb *måtte* according to the norms defined by the Danish language council (Kristensen et al., 2023). These anomalies should also be considered in future studies.

Our study only included one type of task, i.e., proofreading while reading for comprehension. Using eye-tracking, Schotter et al. (2014a) found that the task (proofreading for letter transpositions vs. reading for comprehension) affected processing patterns. The patterns when reading for comprehension may therefore differ from what we find in our study. Still, based on our study, we recommend that future models take the following factors into account, as they may all modulate attention and eye movements:

1. Variation in the type of naturally occurring grammar errors that occur in non-standard language (e.g., syntactic errors compared to morphological errors, and different subtypes within these categories).
2. Variation in error frequencies as a general predictor, and importantly, when present: phonological similarity with the correct form (which tends to be entangled with error frequency).
3. Variation in the reader’s grammatical awareness and proficiency.

## Data availability statement

The dataset for this study and code for analyses can be found in an online repository: http://github.com/ResearchXX/ErrorDetection.

## Ethics statement

The study involving human participants was reviewed and approved by the Faculty of Humanities’ Research Ethics Committee, University of Copenhagen. Written informed consent to participate in this study was provided by participants (if above 18 years old) or by the participants’ legal guardian/next of kin.

## Author contributions

KS and LK contributed to designing the study. KS was responsible for making the test material and collecting data. BI wrote the code for the analyses, which KS used. The first draft was written by KS. LK and BI commented and edited the manuscript. All authors contributed to the article and approved the submitted version.

## Funding

The study was financed by Independent Research Fund Denmark, grant number: 7023-00131B.

## Conflict of interest

The authors declare that the research was conducted in the absence of any commercial or financial relationships that could be construed as a potential conflict of interest.

## Publisher’s note

All claims expressed in this article are solely those of the authors and do not necessarily represent those of their affiliated organizations, or those of the publisher, the editors and the reviewers. Any product that may be evaluated in this article, or claim that may be made by its manufacturer, is not guaranteed or endorsed by the publisher.

## Footnotes

^1^Veldre and Andrews (2018) also argue that the results cannot be reconciled with the alternative *forced fixation account* of preview effects, proposed by Schotter et al. (2014b).

^2^The three education programs (STX, HTX, and HHX) all prepare for higher education, but have different profiles. STX is a general examination program, HTX is a technical examination program with a STEM profile and HHX is a commercial examination program with a business profile (Ministry of Higher Education and Science, 2022).

^3^If p is the probability of detecting an error, 1 − p is the probability of not detecting an error. p/1 − p is the odds of detecting an error and log(p/1 − p) is the logarithm of the odds (logodds).

## Abbreviations

DEF: Definite
INF: Infinitive
N: Neuter
PRS: Present tense
U: Uter (common gender)
