# Supplementary material for: Not all grammar errors are equally noticed: error detection of naturally occurring errors and implications for eye-tracking models of everyday texts
Source: Front Psychol. 2023 Jul 13;14:1124227. doi: 10.3389/fpsyg.2023.1124227 (PMC10373887; doi:10.3389/fpsyg.2023.1124227)
Supplement: Supplementary file 1 [file Data_Sheet_1.docx]

Supplementary Material

[1 Supplementary Figures and Tables 1](#_Toc121856636)

[1.1 Supplementary Tables – model results 1](#_Toc121856637)

[1.2 Supplementary Figures 2](#_Toc121856638)

[2 Stimuli 4](#_Toc121856639)

[2.1 V3 errors 4](#_Toc121856640)

[2.2 Verb errors 6](#_Toc121856641)

[2.3 NP errors 7](#_Toc121856642)

[2.4 Orthographic errors 8](#_Toc121856643)

[3 Test materials 9](#_Toc121856644)

[3.1 Reading task and comprehension questions (in Danish) 9](#_Toc121856645)

[3.2 Questionnaire (in Danish) 12](#_Toc121856646)

[3.3 Questionnaire (in English) 14](#_Toc121856647)

[3.4 Grammar quiz (in Danish) 16](#_Toc121856648)

# Supplementary Figures and Tables

## Supplementary Tables – model results

Table 12. Model (4) estimates for orthographic errors. Dependent variable: Detection (1 = error detected, 0 = error not detected)

| **Random effects** | **Variance** | **Std. Dev.** |  |  |
| --- | --- | --- | --- | --- |
| Participant (Intercept) | 1.0563 | 1.0277 |  |  |
| Item (Intercept) | 0.7147 | 0.8454 |  |  |
| **Fixed effects** | **Estimate** | **Std. Error** | ***z*-value** | ***p*-value** |
| (Intercept) | -5.32930 | 0.67971 | -7.841 | 4.49e-15 *** |
| Type: Reduction of syllable | 1.39987 | 0.54755 | 2.557 | 0.0106 * |
| Type: Compounds written in two | 0.81022 | 0.54737 | 1.480 | 0.1388 |
| Type: Missing silent letter | 0.81582 | 0.54792 | 1.489 | 0.1365 |
| Spelling quiz score | 0.50287 | 0.7715 | 6.518 | 7.11e-11 *** |

Table 13. Model (5) estimates for all error types (collapsed). Dependent variable: Accuracy in percentage

| **Fixed effects** | **Estimate** | **Std. Error** | **t-value** | **p-value** |
| --- | --- | --- | --- | --- |
| (Intercept) | 78.5258 | 1.2311 | 63.787 | < 2e-16 *** |
| Irritation | 1.8184 | 0.3982 | 4.566 | 8.48e-06 *** |

## Supplementary Figures

**Supplementary Figure 1**. Total quiz scores (N of correct answers) and number of detected errors per participant (10 outliers with more false alarms than hits marked with orange)


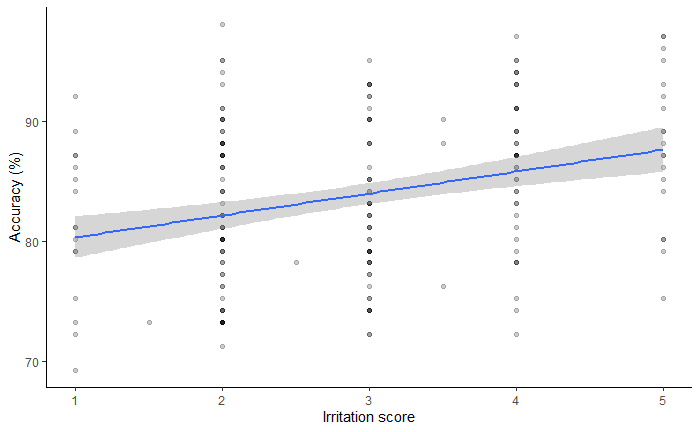


**Supplementary Figure 2**. Effect of reported irritation with language errors on accuracy in error detection (%), model 5.


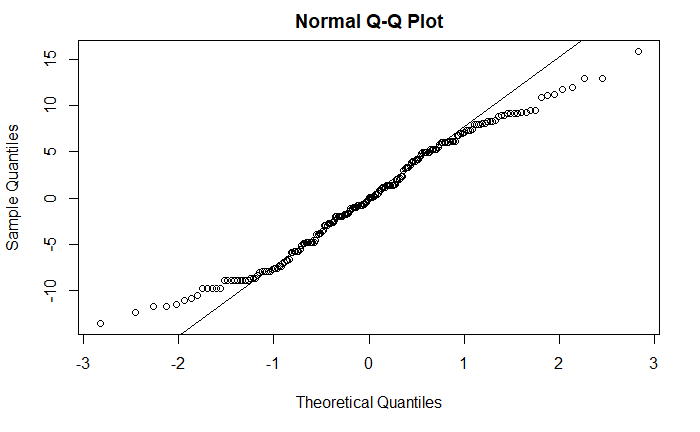


**Supplementary Figure 3**. Normal Q-Q plot of model 5.

# Stimuli

We tried to use controls already present in the texts, or words that could easily be altered from the texts, to avoid making the texts longer and the content even stranger. This means that controls are not perfectly matched to target items. The aim was first to make the controls as similar to targets as possible, with an even distribution in the texts. If more possible control words were available, the ones with a frequency (KorpusDK) closest to the target items were chosen.

## V3 errors

|  |  |  |  |  |
| --- | --- | --- | --- | --- |
| No. | **Adverbial (a, short; b, long)** | ***Subject** | **Finite verb** | **Context** |
| 1a | *Heldigvis* | *han* | *har* | *lavet en aftale:* |
|  | Luckily | he | has | made a deal: |
| 1b | *Til Peters kæmpestore held* |  |  |  |
|  | To Peters great luck |  |  |  |
| 2a | (og) *kl. 14* | *han* | *ankommer* | *til Berlin –* |
|  | (and) at 2 | he | arrives | in Berlin – |
| 2b | (og) *først ud på eftermiddagen* |  |  |  |
|  | (and) first in the afternoon |  |  |  |
| 3a | *Om aftenen* | *han* | *finder* | *sin computer frem.* |
|  | In the evening | he | takes | his computer out |
| 3b | *Omkring kl. 21 om aftenen* |  |  |  |
|  | Around 9 p.m. in the evening |  |  |  |
| 4a | *I spillet* | *man* | *er* | *en ridder, der skal smadre et væmmeligt monster med et gevaldigt hoved.* |
|  | In the game | you | are | a knight, who has to bash a nasty monster with a huge head. |
| 4b | *I det relativt syrede spil* |  |  |  |
|  | In the relatively weird game |  |  |  |
| 5a | *(og) kort efter* | *Peter* | *har* | *det skidt.* |
|  | (and) shortly after | Peter | is | feeling bad. |
| 5b | *(og) allerede efter ganske kort tid* |  |  |  |
|  | (and) already after a fairly short period of time |  |  |  |
| 6a | *I parken* | *han* | *falder* | *til ro igen.* |
|  | In the park | he | calms | down again. |
| 6b | *I den smukke, grønne park* |  |  |  |
|  | In the beautiful, green park |  |  |  |
| 7a | *Lidt derfra* | *dørmanden* | *står* | *og stirrer uhæmmet.* |
|  | A bit from there | the doorman | stands | and stares without restraint. |
| 7b | *Lidt derfra tæt ved udgangen* |  |  |  |
|  | A bit from there, close to the exit |  |  |  |
| 8a | *Pludselig* | *de* | *får* | *øjenkontakt,* |
|  | Suddenly | they | make | eye contact |
| 8b | *Pludselig efter lang tids stirren* |  |  |  |
|  | Suddenly after a long time of staring |  |  |  |
| 9a | *Efter festen* | *de* | *skal* | *på en overdådig bryllupsrejse på et luksuriøst hotel på Maldiverne –* |
|  | After the party | they | are going | on a lavish honeymoon at a luxurious hotel in the Maldives – |
| 9b | *Efter den ekstravagante bryllupsfest* |  |  |  |
|  | After the extravagant wedding |  |  |  |
| 10a | *Ved kiosken* | *børnehaven* | *ligger.* |  |
|  | By the kiosk | the kindergarten | lies. |  |
| 10b | *Ved siden af den lille kiosk* |  |  |  |
|  | Next to the small kiosk |  |  |  |
| 11a | *Derfor* | *Lars* | *har* | *travlt på arbejdet,* |
|  | Therefore | Lars | is | busy at work, |
| 11b | *Derfor og af flere andre grunde* |  |  |  |
|  | Therefore and for multiple other reasons |  |  |  |
| 12a | *Desværre* | *han* | *slipper* | *ikke så let:* |
|  | Unfortunately | he | does | not get off that easily: |
| 12b | *Desværre for den travle mand* |  |  |  |
|  | Unfortunately for the busy man |  |  |  |
| 13a | *På studiet* | *underviserne* | *har* | *fokus på, at de studerende kan beherske mange forskellige genrer:* |
|  | At the university | the teachers | focus | on the students being able to master many different genres: |
| 13b | *På det tidskrævende universitetsstudie* |  |  |  |
|  | During the time-consuming university study |  |  |  |
| 14a | *I dag* | *det* | *driller* | *også.* |
|  | Today | it | is tricky | too. |
| 14b | *Denne ellers hyggelige lørdag formiddag* |  |  |  |
|  | This otherwise pleasant Saturday morning |  |  |  |
| 15a | *Derefter* | *det* | *dur* | *heldigvis igen!* |
|  | Afterwards | it | works | again, fortunately! |
| 15b | *Efter det fantastisk smarte trick* |  |  |  |
|  | After the amazingly clever trick |  |  |  |
| 16a | *Indenfor* | *ekspedienten* | *hilser* | *venligt.* |
|  | Inside | the shop assistant | says hello | with kindness. |
| 16b | *Inde i det hyggelige konditori* |  |  |  |
|  | In the cosy patisserie |  |  |  |

## Verb errors

| **No.** | **HOMOPHONE VERB PAIRS** | | | **HETEROPHONE VERB PAIRS** | | |
| --- | --- | --- | --- | --- | --- | --- |
|  | **Lexeme** | **Characters (N)** | **Frequency**  **(infinitive + present tense)** | **Lexeme** | **Characters (N)** | **Frequency**  **(infinitive + present tense)** |
| 1 | *køre* ‘drive’ | 4 | 14234 | *rejse* ‘travel’ | 5 | 8087 |
| 2 | *motionere* ‘exercise’ | 9 | 120 | *styrketræne* ‘weight-lift’ | 11 | 26 |
| 3 | *fodre* ‘feed’ | 5 | 336 | *passe* ‘look after’ | 5 | 8960 |
| 4 | *føre* ‘guide’ | 4 | 10703 | *lede* ‘lead’ | 4 | 4306 |
| 5 | *sludre* ‘talk’ | 6 | 130 | *chatte* ‘chat’ | 6 | 116 |
| 6 | *gennemføre* ‘complete’ | 10 | 3542 | *afslutte* ‘finish’ | 8 | 769 |
| 7 | *vurdere* ‘evaluate’ | 7 | 5438 | *overveje* ‘consider’ | 8 | 3363 |
| 8 | *præstere* ‘achieve’ | 8 | 705 | *formå* ‘be able to’ | 5 | 894 |
| 9 | *ændre* ‘change’ | 5 | 6526 | *skifte* ‘switch’ | 6 | 3604 |
| 10 | *forklare* ‘explain’ | 8 | 7993 | *berette* ‘recount’ | 7 | 1069 |
| 11 | *angre* ‘regret’ | 5 | 47 | *fortryde* ‘regret’ | 8 | 639 |
| 12 | *manipulere* ‘manipulate’ | 10 | 301 | *fordreje* ‘twist’ | 8 | 67 |
| 13 | *medføre* ‘entail’ | 7 | 3531 | *forårsage* ‘cause’ | 9 | 497 |
| 14 | *score* ‘score’ | 5 | 1177 | *drikke* ‘drink’ | 6 | 3896 |
| 15 | *observere* ‘observe’ | 9 | 253 | *iagttage* ‘watch’ | 8 | 520 |
| 16 | *indikere* ‘indicate’ | 8 | 179 | *antyde* ‘hint’ | 6 | 1145 |
| 17 | *undre* ‘surprise’ | 5 | 2483 | *glæde* ‘please’ | 5 | 5159 |
| 18 | *invitere* ‘invite’ | 8 | 1241 | *indbyde* ‘invite’ | 7 | 307 |
| 19 | *forsvare* ‘defend’ | 8 | 2143 | *beskytte* ‘protect’ | 8 | 2463 |
| 20 | *arrestere* ‘arrest’ | 9 | 115 | *anholde* ‘arrest’ | 7 | 213 |
| 21 | *aktivere* ‘activate’ | 8 | 292 | *tænde* ‘switch on’ | 5 | 1446 |
| 22 | *aflevere* ‘deliever’ | 8 | 1355 | *hente* ‘fetch’ | 5 | 5374 |
| 23 | *skure* ‘scrub’ | 5 | 37 | *skrubbe* ‘scrub’ | 7 | 84 |
| 24 | *lære* ‘learn’ | 4 | 9887 | *skrive* ‘write’ | 6 | 17241 |
| 25 | *sondre* ‘distinguish’ | 6 | 56 | *skelne* ‘distinguish’ | 6 | 1264 |
| 26 | *præsentere* ‘present’ | 10 | 2196 | *fremstille* ‘depict’ | 10 | 2073 |
| 27 | *argumentere* ‘argue’ | 11 | 836 | *kæmpe* ‘fight’ | 5 | 2662 |
| 28 | *fungere* ‘function’ | 7 | 6155 | *virke* ‘work’ | 5 | 9749 |
| 29 | *formatere* ‘format’ | 9 | 5 | *gendanne* ‘restore’ | 8 | 33 |
| 30 | *arrangere* ‘organize’ | 9 | 1136 | *forberede* ‘prepare’ | 9 | 1704 |
| 31 | *brillere* ‘shine’ | 8 | 95 | *optræde* ‘perform’ | 7 | 3720 |
| 32 | *kreere* ‘create’ | 6 | 82 | *bage* ‘bake’ | 4 | 592 |

## NP errors

All target items included regularly inflected adjectives, where inflection is obligatory and not optional (e.g. adjectives ending with -*vis*). Furthermore, we chose adjectives without changes in pronunciation of the stem when the neuter suffix was added, e.g. *sød* [ˈsøˀð] vs. *sødt* [ˈsød̥] ‘sweet’. These restrictions meant that almost half of the target adjectives ended with -*ig*, which might be striking to the participants. Neuter nouns beginning with *d*- or *t*- were avoided, because it might affect participants’ detection of a missing -*t* [d̥] on the adjective that the sound is represented elsewhere, e.g. *et *dejlig tehus* for *et dejligt tehus* ‘a lovely tea house’. The criteria were less strict for the 32 control items (16 uter NPs; 16 neuter NPs); we used inflected adjectives which had not already been used as targets.

| **No.** | **Adjective** | **Neuter noun** | | **Uter noun** | |
| --- | --- | --- | --- | --- | --- |
|  |  | **Lexeme** | **Characters (N)** | **Lexeme** | **Characters (N)** |
| 1 | *dejlig* ‘lovely’ | *kæledyr* ‘pet’ | 7 | *undulat* ‘budgie’ | 7 |
| 2 | *enorm* ‘huge’ | *kinderæg* ‘Kinder Egg’ | 8 | *slikpose* ‘bag of sweets’ | 8 |
| 3 | *fin* ‘nice’ | *center* ‘mall’ | 6 | *butik* ‘store’ | 5 |
| 4 | *brun* ‘brown’ | *bælte* ‘bælte’ | 5 | *rygsæk* ‘rucksack’ | 6 |
| 5 | *lækker* ‘smashing’ | *hotelværelse* ‘hotel room’ | 12 | *lejlighed* ‘apartment’ | 9 |
| 6 | *mærkelig* ‘weird’ | *univers* ‘universe’ | 7 | *verden* ‘world’ | 6 |
| 7 | *solid* ‘solid’ | *morgenmåltid* ‘morning meal’ | 12 | *morgenmad* ‘breakfast’ | 9 |
| 8 | *utrolig* ‘unbelievable’ | *held* ‘luck’ | 4 | *chance* ‘opportunity’ | 6 |
| 9 | *hård* ‘tough’ | *løb* ‘run’ | 3 | *dag* ‘day’ | 3 |
| 10 | *stærk* ‘strong’ | *bolsje* ‘hard candy’ | 6 | *lakrids* ‘liquorice’ | 7 |
| 11 | *lokal* ‘local’ | *sted* ‘place’ | 4 | *bar* ‘bar’ | 3 |
| 12 | *iskold* ‘ice-cold’ | *shot* ‘shot’ | 4 | *drink* ‘drink’ | 5 |
| 13 | *ren* ‘clean’ | *bord* ‘table’ | 4 | *stol* ‘chair’ | 4 |
| 14 | *hvid* ‘white’ | *stearinlys* ‘candle’ | 10 | *olielampe* ‘oil lamp’ | 9 |
| 15 | *venlig* ‘friendly’ | *smil* ‘smile’ | 4 | *gestus* ‘gesture’ | 6 |
| 16 | *uforglemmelig* ‘unforgettable’ | *arrangement* ‘function’ | 11 | *begivenhed* ‘event’ | 10 |
| 17 | *vanvittig* ‘insane’ | *antal* ‘number’ | 5 | *mængde* ‘amount’ | 6 |
| 18 | *rummelig* ‘spacious’ | *hus* ‘house’ | 3 | *villa* ‘villa’ | 5 |
| 19 | *grøn* ‘green’ | *areal* ‘area’ | 5 | *park* ‘park’ | 4 |
| 20 | *skøn* ‘great’ | *barn* ‘child’ | 4 | *dreng* ‘boy’ | 5 |
| 21 | *frygtelig* ‘terrible’ | *kaos* ‘chaos’ | 4 | *uro* ‘stir’ | 3 |
| 22 | *dyr* ‘expensive’ | *system* ‘system’ | 6 | *løsning* ‘solution’ | 7 |
| 23 | *vidunderlig* ‘wonderful’ | *arbejde* ‘job’ | 7 | *kæreste* ‘girlfriend’ | 7 |
| 24 | *lykkelig* ‘happy’ | *ægtepar* ‘married couple’ | 7 | *familie* ‘family’ | 7 |
| 25 | *lang* ‘long’ | *indlæg* ‘article’ | 6 | *artikel* ‘article’ | 7 |
| 26 | *ubehagelig* ‘uncomfortable’ | *forløb* ‘process’ | 6 | *affære* ‘affair’ | 6 |
| 27 | *besværlig* ‘difficult’ | *program* ‘program’ | 7 | *proces* ‘process’ | 6 |
| 28 | *elendig* ‘lousy’ | *product* ‘product’ | 7 | *opfindelse* ‘invention’ | 10 |
| 29 | *fabelagtig* ‘fabulous’ | *uddrag* ‘extract’ | 6 | *novelle* ‘short story’ | 7 |
| 30 | *smuk* ‘beautiful’ | *slør* ‘veil’ | 4 | *kjole* ‘dress’ | 5 |
| 31 | *hel* ‘whole’ | *vindue* ‘window’ | 6 | *sektion* ‘section’ | 7 |
| 32 | *saftig* ‘juicy’ | *rosinbrød* ‘raisin bread’ | 9 | *frugtkage* ‘fruit cake’ | 9 |

## Orthographic errors

| **Error** | **Target form** | **Translation** |
| --- | --- | --- |
| **Missing double consonants** | | |
| *startskudet* | *startskuddet* | ‘the starting signal’ |
| *parets* | *parrets* | ‘the couple’s’ |
| *kroniker* | *kronikker* | ‘feature articles’ |
| *butiken* | *butikken* | ‘the shop’ |
| *girafen* | *giraffen* | ‘the giraffe’ |
| **Split compounds** | | |
| *æble cider* | *æblecider* | ‘apple cider’ |
| *by vandring* | *byvandring* | ‘city walk’ |
| *drømme bryllup* | *drømmebryllyp* | ‘dream wedding’ |
| *chokolade smag* | *chokoladesmag* | ‘chocolate taste’ |
| *prøve smagning* | *prøvesmagning* | ‘sample tasting’ |
| **Missing silent letters** | | |
| *egenlig* | *egentlig* | ‘actually’ |
| *sjælent* | *sjældent* | ‘rarely’ |
| *(i det) minste* | *(i det) mindste* | ‘at least’ |
| *siste* | *sidste* | ‘last’ |
| *selsikre* | *selvsikre* | ‘confident’ |
| **Reduced syllables** | | |
| *temlig* | *temmelig* | ‘rather’ |
| *endlig* | *endelig* | ‘finally’ |
| *spør* | *spørger* | ‘ask’ (present tense) |
| *virklig* | *virkelig* | ‘really’ |
| *ærgligt* | *ærgerligt* | ‘annoyingly’ |

# Test materials

## Reading task and comprehension questions (in Danish)

Errors are marked with colors: V3 errors, verb errors, NP errors and orthographic errors.

Peter i Berlin

Peter er 33 år og en ivrig maratonløber. I dag er en vigtig dag. Han køre til Tyskland, nærmere bestemt Berlin. Her skal han deltage i et populært løb.

Peter er typen, der sætter ambitiøse mål for alt. Han skal styrketræner meget og gerne hver dag. Han risikerer gerne liv og helbred for en rigtig udfordring, og han vil sejre for enhver pris. Imidlertid har han også en blød side: Han er en rigtig dyreven! Peter har en dejligt kæledyr, som ærgligt nok ikke kan komme med til Berlin.

Heldigvis han har lavet en aftale: Naboens børn passe Piphans, som fuglen hedder. Som tak for nabobørnenes hjælp vil han købe en enormt slikpose med hjem.

Peter tager afsted tidligt om morgenen, og først ud på eftermiddagen han ankommer til Berlin – dagen inden løbet. Stemningen i byen er helt i top.

Peter har tid til lidt sightseeing, så han har meldt sig til en by vandring. Guiden vil fører turisterne gennem det 19.000 m^2^ store mindesmærke for Europas myrdede jøder, som består af 2.711 betonpiller. De når også at gå forbi et fin center, hvor Peter bl.a. køber et brun rygsæk. Peter har booket en lækkert lejlighed i centrum. Omkring kl. 21 om aftenen han finder sin computer frem. Han vil chatter med en gammel ven over Skype. Vennen fortæller, at der er kommet et sjovt computerspil fra deres yndlingsspiludviklere, og Peter downloader det straks! Spillet foregår i en mærkeligt univers med prinsesser og zombier. I spillet man er en ridder, der skal smadre et væmmeligt monster med et gevaldigt hoved. Peter gennemføre første bane. Det bliver desværre sent. Peter spiser også et stort marcipanbrød og slubrer et par dåser æble cider og øl i sig. Det er ikke så godt.

Næste morgen er Peter ikke helt frisk. Han overveje situationen: Kan han levere et godt resultat i dag? Eller er det egenlig bedre at droppe at løbe? Han bliver så skuffet, hvis han ikke kan præsterer at få en god tid. Efter et solid morgenmåltid beslutter han sig for at løbe.

Startskudet lyder, og allerede efter ganske kort tid Peter har det skidt. Dagsformen er ikke god, og han kan ikke holde et højt tempo, som han plejer. Han må skifter taktik. Et sted i starten af ruten kan man skyde genvej gennem en smal sidegade. Sikke et utrolig chance!

Peter kommer i mål med en bedre tid, end han plejer. Men nogen har set ham snyde. Peter er opdaget, og han forklare det hele, da de løbsansvarlige spør. Han burde angrer det, han har gjort. Sikke en åndssvag bommert.

Han håber godt nok ikke, at det kommer frem i medierne – de kan fordrejer alt. Det ville være temlig pinligt, hvis kammeraterne i løbeklubben derhjemme fik noget at vide! Peter går hen og sætter sig i en park for at samle tankerne. I parken han falder til ro igen. I det minste har han lært noget vigtigt: Uærlighed medføre mange problemer.

Efter en hårdt dag vil Peter tage i byen. Først spiser han lige en stærkt bolsje for at tage noget af den dårlige ånde. Han orker ikke proppede turistfælder, så han finder et lokal bar. Ifølge Peter og hans venner er det ikke en ordentlig bytur, hvis man ikke kan drikker løs.

Han går op i baren og bestiller en iskoldt drink. Bardisken er lidt for klistret for Peters smag, så han forsøger at finde en rent bord. Mens han kigger, får han øje på noget interessant: en ældre, men ret flot, tysk kvinde. Peter sætter sig. Han iagttage hende fra sin plads og tænker, at hun faktisk ser rigtig flot ud, som hun sidder der i lyset fra et hvid stearinlys. Peter opdager, han ikke er den eneste, der kigger på hende. Lidt derfra tæt ved udgangen dørmanden står og stirrer uhæmmet.

Peter må rykke snart. Han vil indikerer, at han er interesseret. Den selsikre Peter tænker, at hun undre sig over, at en ung mand er interesseret.

Pludselig de får øjenkontakt, og kvinden sender Peter en venligt gestus. Nu tør Peter godt gå derhen! Han tager en siste tår og går hen mod hende …

Lars og Lone i Hvidovre

Der sker store ting for Lars og Lone. Efter at have sparet op i årevis er tiden endlig kommet: Det er i år, de indbyde hele familien til bryllup. Det bliver et uforglemmelig begivenhed, for Lone kan organisere de vildeste fester. Lars og Lone elsker fransk champagne på elegante flasker, og de har allerede indkøbt en vanvittigt antal. Efter festen de skal på en overdådig bryllupsrejse på et luksuriøst hotel på Maldiverne – dét ser de frem til!

Lone og Lars bor sammen med sønnen Storm i et rummelig hus i Hvidovre. Kvarteret er hyggeligt, med mange, gode faciliteter – fx en herlig legeplads, et grøn park og en kiosk.

Ved siden af den lille kiosk børnehaven ligger. Her går Storm. Han er altid glad, og pædagogerne i børnehaven synes, han er en skønt dreng.

Langt størstedelen af tiden plaprer Storm bare løs om girafen Ingolf – et gammelt tøjdyr, han fandt i glemmekassen. Lars er ansat i PET, som lige nu skal forsvarer staten mod et stort cyberangreb, der har skabt et frygtelig kaos i hele samfundet. Derfor Lars har travlt på arbejdet, men de er heldigvis langt i sagen.

Politiet, som lige nu skal patruljere i civil i et skummelt område, regner med, at de kan anholder den første mistænkte, inden ugen er omme. Lars går meget op i sikkerhed og har installeret et nyt alarmsystem i parets hus. Han tænde alarmen, hver gang de forlader huset – også hvis de bare går en hurtig tur i villakvarteret. Det synes Lone er åndssvagt.

Hun synes også, det er åndssvagt at bruge så mange penge på alarmer – det var en dyrt system. De penge kunne have været brugt på det kommende drømme bryllup! Normalt styrer Lone ellers familiens økonomi med hård hånd, og hun vil notere alle større udgifter i et særligt regneark. Lars vil hellere sikre huset end at bruge penge på et tåbeligt champagnespringvand til brylluppet.

Lars synes, han har et vidunderlig kæreste, selvom Lone er lidt sur over, at han arbejder så meget. Hun synes, Lars skal prioritere familien højere. Hun synes heller ikke, han hjælper nok til derhjemme, så Lars lover, at han aflevere Storm på mandag. Desværre for den travle mand han slipper ikke så let: Lone svarer, at hun først bliver tilfreds, hvis Lars også vil skurer gulvet, inden Lones mor kommer på besøg om aftenen. Ellers er de nu en lykkeligt familie det meste af tiden.

Lone har også travlt med mange ting. Hun læser journalistik på RUC. Hun skal skriver en masse i løbet af et semester. På det tidskrævende universitetsstudie underviserne har fokus på, at de studerende kan beherske mange forskellige genrer: Man sondre mellem mange forskellige typer artikler. Alt fra baggrundsartikler, ledere, features, kroniker, reportager og klummer til interviews.

Lone synes ikke, det er nemt. Hun arbejder på en langt indlæg, som gerne skal udkomme i et nyt magasin. Artiklen handler om Lones nabo, Monika, som påstår, hun har været udsat for chikane på sin arbejdsplads og er blevet fyret uden grund. Lone vil præsenterer Monika som en troværdig kilde. Det baserer Lone bl.a. på, at man under retssagen ser, hvor godt Monika kæmpe for sin sag. Lone kan også mærke på Monika, at hun virklig synes, det har været et ubehagelig affære.

I de ellers tiltrængte weekender sidder Lone tit og producerer eller omstrukturerer sine tekster, men der er ofte problemer med Word. Det er et besværlig program. Lone synes, det er lidt for sjælent, at programmet fungere ordentligt. I dag det driller også.

Hun skal gendanner dokumentet. Efter det fantastisk smarte trick det dur heldigvis igen! Lone (og faktisk også Lars) synes i dén grad, at Word er en elendigt produkt.

Lone er også med i en aktiv gruppe på studiet, hvor de forberede en hyggelig aften med oplæsning af skønlitteratur. Lone kan allerede se for sig, hvordan hun vil brillerer med et af sine egne værker. Hun synes selv, hun har skrevet et fabelagtig uddrag. Inden Lone er færdig med sine dagdrømmerier, afbryder Lars. Han skal altid forstyrre.

De skal gå nu, hvis de skal nå til prøve smagning af bryllupskager hos byens bedste konditor, som bage nogle helt ekstravagante kager, og som har vundet mange priser. Og Lars skal jo først lige nå at slå alarmen til!

På vej hen til konditoriet ser Lone et smuk kjole i en eksklusiv brudebutik. Lars er glad for, at butiken er lukket i dag, så de kan komme hurtigt videre. Allerede på meget lang afstand kan de se et hel vindue med de yndigste bryllupskager. Indenfor ekspedienten hilser venligt. Hun serverer fire slags kage for dem. Lone synes, det er et vigtigt valg, de skal træffe. Lars vil faktisk hellere hjem og ligge på sofaen, så han stemmer bare på chokolade smag, og Lone er heldigvis enig.

I konditoriet er der mange lækre fristelser, og de køber også en saftigt frugtkage til at tage med hjem. Storm kan næsten ikke gå efter at have spist så meget kage!

**Spørgsmål til teksterne**

**Peter i Berlin**

Sæt kryds ved det rigtige svar:

1 *Skal Peter til Berlin for at løbe maraton? ja nej*

2 *Bliver Peters snyderi opdaget? ja nej*

3 *Tager Peter i Berlin Zoo? ja nej*

**Lars og Lone i Hvidovre**

Sæt kryds ved det rigtige svar:

1 *Har* *Lars og Lone en datter? ja nej*

2 *Arbejder Lars for PET? ja nej*

3 *Læser Lone til tandlæge? ja nej*

## Questionnaire (in Danish)

**Spørgeskema**

**A. Generel information**

A1 Hvilket køn har du? Sæt kryds: mand kvinde andet

A2 Hvor gammel er du? Sæt kryds: 17 år 18 år 19 år andet: ____ år

A3 Hvilken studieretning har du? Sæt kryds:

STX: naturvidenskab samfundsvidenskab sprog kunst

HTX: anvendt naturvidenskab teknologi kommunikationsteknik

HHX: økonomi og marked økonomi og sprog sprog

**B. Sproglig baggrund**

A1 Er du ordblind? nej ja måske (men jeg er ikke blevet testet)

A2 Hvilke sprog taler du, hvornår begyndte du ca. at lære dem, og hvor godt taler du dem? Husk at nævne, hvornår du begyndte at lære dansk – var det fx som barn eller senere i livet?

| **Hvilke sprog taler du?** | **Hvor gammel var du ca., da du startede med at lære sproget?** *Fx ”0 år”* | **Hvor godt taler du sproget?** *Fx ”flydende”, ”avanceret niveau”, ”mellem”, ”begynder”* | **Taler du normalt sproget med din mor eller far derhjemme?**  *”Ja”/”nej”* |
| --- | --- | --- | --- |
| Dansk |  |  |  |
| Engelsk |  |  |  |
|  |  |  |  |
|  |  |  |  |
|  |  |  |  |
|  |  |  |  |

A3 Har du tidligere boet i andre kommuner (eller lande), end hvor du bor nu?

nej ja

Hvis ja: Hvor? Hvor længe boede du der, og hvor gammel var du ca., mens du boede der? Skriv svarene i tabellen:

| **Kommune eller land** | **Hvor længe boede du der?** (Hvor mange måneder/år?) | **Hvor gammel var du ca., mens du boede der?** |
| --- | --- | --- |
|  |  |  |
|  |  |  |
|  |  |  |
|  |  |  |

**C. Holdning til sprogfejl**

C1 Hvor irriteret bliver du, hvis der er sprogfejl (dvs. stavefejl og grammatikfejl) i en tekst, du læser? Markér dit svar ved at sætte en ring om én af nedenstående bokse.

| *slet ikke irriteret* | *en smule irriteret* | *irriteret* | *meget irriteret* | *ekstremt irriteret* |
| --- | --- | --- | --- | --- |

C2 Her kan du uddybe dit svar. Kommer det fx an på, hvilken type tekst der er tale om? Er der fx nogle særlige sprogfejl, du især bliver irriteret over? Eller er du helt ligeglad med, om der er sprogfejl i en tekst?

_____________________________________________________________________________________________________________________________________________________________________________________________________________________________________________________________________________________________________________________________________________________________________________________________________________________

## Questionnaire (in English)

**Questionnaire**

**A. General information**

A1 What is your gender? Check the box: man woman other

A2 How old are you? Check the box: 17 y/o 18 y/o 19 y/o other: ____ y/o

A3 What is your study program? Check the box:

STX: natural science social science language art

HTX: applied natural sciences technology communication and IT

HHX: economy and market economy and language language

**B. Linguistic background**

A1 Are you dyslexic? no yes maybe (but I haven’t been tested)

A2 Which languages do you speak, approximately when did you start to learn them, and how well do you speak them? Remember to mention when you started learning Danish – e.g. was it as a child or later in life?

| **Which languages do you speak?** | **How old were you approximately, when you started learning the language?** *E.g. ”0 y/o”* | **How well do you speak the language?** *E.g.”fluently”, ”advanced level”, ”intermediate”, ”beginner”* | **Do you normally speak the language with your mom or dad at home?**  *”Yes”/”no”* |
| --- | --- | --- | --- |
| Danish |  |  |  |
| English |  |  |  |
|  |  |  |  |
|  |  |  |  |
|  |  |  |  |
|  |  |  |  |

A3 Have you previously lived in other municipalities (or countries) than where you currently reside?

no yes

If yes: Where? How long did you live there, and how old were you approximately, while you lived there? Write your answers in the table:

| **Municipality or country** | **How long did you live there?** (How many months/years?) | **How old were you approximately, while you lived there?** |
| --- | --- | --- |
|  |  |  |
|  |  |  |
|  |  |  |
|  |  |  |

**C. Attitude to language errors**

C1 How annoyed are you, if there are language errors (i.e. spelling mistakes and grammar mistakes) in a text you are reading? Mark your answer by circling one of the boxes below.

| *not at all annoyed* | *a bit annoyed* | *annoyed* | *very annoyed* | *extremely annoyed* |
| --- | --- | --- | --- | --- |

C2 Here, you can elaborate on your answer. Does it depend on what type of text it is? Are there certain types of language mistakes that you are particularly annoyed by? Or are you completely indifferent to the presence of language errors in a text?

_____________________________________________________________________________________________________________________________________________________________________________________________________________________________________________________________________________________________________________________________________________________________________________________________________________________

## Grammar quiz (in Danish)

**Grammatikquiz**

**FORMÅL:** Nogle af spørgsmålene i denne quiz kan måske virke nemme: Vi skal bare tjekke, om du kender helt almindelig dansk grammatik. Andre spørgsmål er måske lidt sværere. Det er vigtigt, at du ikke slår svaret op i en ordbog eller på nettet. Vi er nemlig interesserede i, hvad du gør, når du ikke har mulighed for at slå noget op.

**1 Ordstilling**

Her skal du indsætte ”*han ankommer til Berlin*” på linjerne, sådan at ordene kommer til at stå i rigtig rækkefølge.

1.1 *Kl. 14 n*

1.2 *Og g*

1.3 *Hvis alt går godt, n*

**2 Adjektiver og artikler**

Indsæt den korrekte form af tillægsordet (adjektivet) *lækker* på nedenstående linjer

2.1 *en _________________ kage*

2.2 *et _________________ brød*

Hedder det *en* eller *et*? Sæt kryds:

2.3 *en et* *shot*

2.4 *en et* *undulat*

2.5 *en et bolsje*

2.6 *en et lakrids*

**3 Verber**

Sæt kryds ved den korrekte form af verbet:

3.1 *Nu ______ han hurtigt. køre kører*

3.2 *Han vil ______ hurtigt. køre kører*

3.3 *Hun skal ______ i morgen. rejse rejser*

3.4 *Hun ______ senere i dag, hvis piloterne ikke strejker. rejse rejser*

3.5 *Han ______ mange nyttige ting. lære lærer*

3.6 *Han skal ______ at danse brudevals inden brylluppet. lære lærer*

3.7 *De skal ______ ekstreme mængder kage i weekenden.*  *spise spiser*

3.8 *Om lidt ______ de en sandwich. spise spiser*

**4 Stavning**

Er disse ord stavet korrekt? Hvis ikke, så skriv den korrekte form:

| 4.1 | *butiken* | korrekt | Nej, det staves: | ________________________ |
| --- | --- | --- | --- | --- |
| 4.2 | *by vandring* | korrekt | Nej, det staves: | ________________________ |
| 4.3 | *virkelig* | korrekt | Nej, det staves: | ________________________ |
| 4.4 | *æblecider* | korrekt | Nej, det staves: | ________________________ |
| 4.5 | *sjælent* | korrekt | Nej, det staves: | ________________________ |
| 4.6 | *temlig* | korrekt | Nej, det staves: | ________________________ |
| 4.7 | *startskuddet* | korrekt | Nej, det staves: | ________________________ |
| 4.8 | *egentlig* | korrekt | Nej, det staves: | ________________________ |
